# Statins Inhibit Fibrillary *β*-Amyloid Induced Inflammation in a Model of the Human Blood Brain Barrier

**DOI:** 10.1371/journal.pone.0157483

**Published:** 2016-06-16

**Authors:** Jarred M. Griffin, Dan Kho, E. Scott Graham, Louise F. B. Nicholson, Simon J. O’Carroll

**Affiliations:** 1 Centre for Brain Research, School of Medical Sciences, Faculty of Medical and Health Sciences, University of Auckland, Auckland, New Zealand; 2 Department of Anatomy and Medical Imaging, School of Medical Sciences, Faculty of Medical and Health Sciences, University of Auckland, Auckland, New Zealand; 3 Department of Pharmacology and Clinical Pharmacology, School of Medical Sciences, Faculty of Medical and Health Sciences, University of Auckland, Auckland, New Zealand; Hungarian Academy of Sciences, HUNGARY

## Abstract

**Background:**

Astrocytes and cerebral endothelial cells are important components of the blood-brain barrier (BBB). Disruption to this barrier through inflammation is a major contributor to Alzheimer’s disease (AD) pathology. The amyloid beta (A*β*) protein is known to exist in several forms and is a key modulator of AD that is known to cause inflammation and changes to BBB function. While one of these forms, fibrillary A*β* (fA*β*), is known to cause endothelial cell death at the BBB, no studies have looked specifically at its role on inflammation in a model of the human BBB.

**Aims:**

To determine if fA*β* is inflammatory to the human BBB. As statins have been shown to be anti-inflammatory and protective in AD, we also tested if these could inhibit the inflammatory effect of fA*β*.

**Methods:**

Using cultured cerebral endothelial cells and astrocytes we determined changes in cytokine release, cell toxicity and barrier function in response to fibrillary *β*-amyloid_1–42_ (fA*β*_1–42_) alone and in combination with statins.

**Results:**

fA*β*_1–42_ induced inflammatory cytokine release from endothelial cells in the absence of cell toxicity. It also induced astrocyte cytokine release and cell death and caused a loss of barrier integrity. Statin treatment inhibited all of these effects.

**Conclusions:**

We conclude that fA*β*_1–42_ has both inflammatory and cytotoxic effects on the BBB and the protective effect of statins in AD may in part be through inhibiting these effects.

## Introduction

In neurodegenerative disease such as Alzheimer’s disease (AD), the most common form of dementia, deleterious chronic inflammation is an important player [1]. A better understanding of the processes that precede and result from this inflammation will provide insights into ways to combat the disease. We have investigated one vascular aspect of neurodegeneration which focuses on the blood-brain barrier (BBB) in order to better understand how this barrier is affected by disease conditions. There is mounting evidence to suggest that the components of the BBB can respond to inflammation, which could in turn potentially contribute to events leading to subsequent neurodegeneration and may also potentiate the neuroinflammatory cycle. The BBB is a unique anatomical structure that is essential for maintaining homeostasis of the human brain parenchymal microenvironment [2]. Cerebral endothelial cells and astrocytes are among the key players in the human brain inflammatory response, initiated by inflammatory events in the brain’s environment. Astrocytes are complex, highly differentiated cells that are present throughout the entire CNS and may be activated in response to a wide variety of inflammatory stimuli changing their morphology and molecular expression accordingly [2, 3]. The cells of the BBB are highly responsive to the inflammatory processes and can be modulated by inflammatory mediators of both the systemic and central nervous systems. A major consequence of this inflammation is the loss of barrier integrity. In health, the BBB represents a highly selective barrier that prevents entry of cells, bacteria and viruses, but allows selective passage of water, gases and certain molecules that are crucial to neural functions. However, in pathology such as AD, many pro-inflammatory mediators such as tumour necrosis factor alpha (TNF*α*) or interleukin 1 beta (IL-1*β*) cause loss of ‘tightness’ that increases BBB permeability [4]. Increased BBB permeability allows immune cells to enter the brain parenchyma and worsen pathology. Relapsing remitting multiple sclerosis is a prime example of this where auto-reactive immune cells breach the BBB and cause central nervous system (CNS) lesions [5]. There is a link between amyloid beta (A*β*), cytokine release, the BBB and AD progression. For example, amyloid-beta 1–42 (A*β*_1–42_) activates the inflammatory toll-like receptors [6] and receptors for advanced glycation end products [7], and binds the complement factor C1 activating the classical complement pathway of cytotoxicity [8] and leading to cytokine secretion. Cytokines may also increase the production of A*β*. For example, the inflammatory cytokine IL-8 has been shown to increase beta-secretase 1 (BACE-1), amyloid precursor protein (APP) processing and A*β* production in SH-SY5Y neuroblastoma cells [9]. Moreover, inflammatory events and redox insults can lead to increased production of toxic A*β* peptides [10]. While an acute inflammatory episode is crucial for insults, a prolonged, chronic cycle of inflammation creates a toxic environment of reactive oxygen species and phagocytic immune cells that diverges from healthy homeostasis towards AD progression. A*β* is known to exist in three different states; monomer, oligomer and fibrillary. All forms of A*β* have been shown to have an effect at the BBB in both rodents and humans, leading to a change in BBB permeability and cell viability [11–14]. While all cause changes at the BBB, the different forms of A*β* have been shown to have differential effects. Monomeric and oligomeric A*β* are pro-inflammatory at the BBB, with the oligomeric form being the most inflammatory and both forms lead to changes in BBB and cell viability. In contrast, fibrillary A*β* (fA*β*)affects the BBB to lesser extent but has been shown the high concentrations are toxic to endothelial cells of the BBB [15]. fA*β* has been shown to induce inflammation in rat astrocyte [16] cultures but to date, few studies have looked at the inflammatory effects of fA*β* on cerebral endothelial cells and no studies have looked at it specifically in the human BBB. Drugs with anti-inflammatory properties have become the focus of neurodegenerative disease research based on the rationale that they could dampen down inflammatory events that are a consequence of the pathology and/or events that precede the pathology. Statins have previously been shown to reduce BBB permeability and restrict leukocyte migration in BBB-derived endothelial cells in a number of models of disease [17–23]. The statin drugs inhibit HMG-CoA reductase, which forms the rate-limiting step of de novo cholesterol biosynthesis. Statin drugs are reported to have potent anti-inflammatory properties [24–26] and there is some evidence that they are protective against AD [27–29]. Studies have demonstrated that statins can inhibit the inflammatory effects of A*β* on endothelial cells [30] but they have not looked specifically at whether statins can inhibit the effects of fA*β*. fA*β* is known to be a major contributor to BBB damage in AD [15, 31–33] so determining if statins can target the effects of fA*β* will provide some insight into their possible role in preventing AD progression. The aims of this study were to determine whether fA*β* can have inflammatory effects on endothelial cells and astrocytes of the BBB and whether statin drugs are protective against these inflammatory effects in a co-culture model of the human BBB.

## Materials and Methods

### Cell Culture

This study used human cell cultures of astrocytes and brain microvascular endothelial cells. The NT2/A astrocytes are derived from the retinoic acid differentiation of the NT2/D1 teratocarcinoma cells. These cells have been characterised and have a cytokine profile similar to primary astrocytes and other astrocyte cell lines [34]. The human cerebral microvascular endothelial (hCMVEC) cells were purchased from Applied Biological Materials (ABM) Inc, Canada (cat # T0259). We have extensively characterised the endothelial phenotype of this cell line in terms of its barrier resistance, cytokine secretion and cell surface adhesion molecules [35].

### Reagents

Cell culture plasticware was purchased from Corning. All cell culture media and additives where purchased from Life Technologies except fetal bovine serum, which was purchased from Moregate Biotech. All-trans retinoic acid, uridine, 5-fluorodeoxyuridine and arabinofuranosyl were purchased from Sigma. The A*β*_1–42_ and A*β*_42–1_ fragments were purchased from Bachem and lovastatin and simvastain were purchased from Cayman Chemicals.

### NT2-A astrocytes

Human neuron-committed teratocarcinoma (NT2) cells were differentiated by using a method previously described [36]. One day prior to experimentation cells were resuscitated from cryopreservation and plated at a density of 1.25x10^4^ cells/well in a 96 well plate or 24 well cell culture insert (3.0 *μ*m pore size) or 7x10^4^ cells/well in a 24 well plate. The cells were plated in DMEM/F12, supplemented with 10% FBS and 1% GlutaMAX, and maintained in a humidified atmosphere at 37°C with 95% air/5% CO_2_.

### hCMVEC endothelial cells

The hCMVEC endothelial cell line was used between passage 7 and 20. Cells were maintained in M199 medium, supplemented with 10% FBS (Invitrogen), 1 *μ*M hydrocortisone, 3 ng/mL hFGF, 10 ng/mL hEGF, 10 *μ*g/mL heparin and 80 *μ*M cAMP in a humidified atmosphere at 37°C with 5% CO_2_. For cytometric bead array, western blotting and MTT assays cells were plated at a density of 1 x 10^5^ cells/well in a 24 well plate or 1 x10^4^ cells/well in a 96 well plate in M199 plating media supplemented with 2% FBS, 1 *μ*g/mL hydrocortisone, 1 *μ*M insulin and 80 *μ*M cAMP and treated 24 hours later. For ECIS and EVOM TEER experiments cells were plated a density of 2 x 10^4^ cells/well in a 96W20dfi ECIS array plate or cell culture insert in 10% FBS M199 plus additives. 24 hours prior to treatment media was changed to 2% FBS M199 media plus additives.

### *β*-Amyloid_42–1_ treatments

Fresh A*β*_1–42_ and control A*β*_42–1_ peptide stock solutions were prepared at 1 mg/mL in DMSO. Aliquots were added to culture medium at twice the experimental concentration (2 *μ*M) and maintained at 37°C for 5 days. Following incubation, the presence of aggregates was determined using microscopy (S1 Fig). The solution was then centrifuged at 14,000 rpm, 4°C for 10 min to pellet the aggregates and the supernatant containing monomeric and and oligomeric A*β* was removed [12]. The aggregates were resuspended and peptide solutions were then applied to the cells at 1 *μ*M in the respective culture medium. To specifically look at inflammatory effects on endothelial cells, a concentration of 1 *μ*M was used as it has been reported previously that A*β*_1–42_ concentrations between 0.5 *μ*M and 5 *μ*M produce similar inflammatory effects [37] and higher concentrations of fA*β* are toxic to cerebral endothelial cells [15].

### Statin drug treatments

Simvastatin and lovastatin were applied to the cells at a concentration of 0.5 *μ*M. Treated cells were incubated in culture media as previously specified a humidified atmosphere at 37°C with 5% CO_2_.

### hCMVEC/NT2/A co-cultures

NT2/A cells were plated on the bottom of 0.1% gelatin-coated 24-well cell culture inserts and incubated for 4–5 hours. Once the NT2/A cells had adhered, inserts were placed upright into 24 well plates, containing 900 *μ*l of 10% FBS DMEM. hCMVECs were then plated on to the top of the insert in 10% FBS M199 plus additives. Twenty four hours after plating of the hCMVEC cells, 1 *μ*M fA*β*_1–42_ was added to the 24 well plate (astrocyte side) and 0.5 *μ*M of the respective statin was added to the well insert (endothelial side).

### Barrier Integrity measurements using ECIS ZΘ TEER technology

ECIS experiments were conducted using 96W20dfi ECIS arrays. Following array preparation, the hCMVECs were seeded at 2 x 10^4^ cells/well. Cells were typically treated around 48 hours after seeding, which was determined previously as the period where the resistance level had stabilised (900–1000 Ω). Following drug addition, the ECIS experiments were continuously monitored for 2–3 days to capture both acute and longer term changes in resistance.

### EVOM TEER measurements

Transendothelial electrical resistance (TEER) measurements were carried out using the EVOM2 Voltohmmeter and STX3 electrode. Co-cultures were set up as described above. TEER readings were taken every 24 hours. A blank reading (coated insert but no cells) was also carried out to allow calculation of actual TEER values. On day 4, media was replaced with 2% FBS M199 (endothelial side) and 2% FBS DMEM (astrocyte side). Media was then replaced every 2 days. Treatment with fA*β*_1–42_ or vehicle was carried out on day 9, once the TEER reading had stabilised (TEER readings stabilised at ~60 Ω cm^2^). Media containing fA*β*_1–42_ or vehicle was changed every 2 days.

### Multiplexed cytometric bead array (CBA)

The cytometric bead array (CBA; BD Biosciences; see http://www.bdbiosciences.com/CBA) method used was the same as that previously described [34]. Soluble cytokines were measured by multiplexed CBA. The cytokines assayed in this study were based on the cytokine expression profile of the hCMVEC and NT2/A cells as described previously [34, 35]. Endothelial cells were plated at 1 x 10^5^ cells/well and NT2/A cells at 7 x 10^4^cells/well in a 24 well plate for mono-culture experiments or as co-cultures as described above. For co-culture experiments, media was removed 48 hours after addition of fA*β*_1–42_ and statins. CBA samples were analysed using the BD Accuri C6 (BD Biosciences). Data were analysed using FCAP Array software (version 3.1) that automatically converts the sample mean fluorescent intensity values to pg/mL concentration based on the standard curve. Following generation of the standard curves for each cytokine, data was included where the R^2^ value was greater than 99.8, the sample concentrations were determined from the mean fluorescence intensity (MFI) for samples that were within the range of the standard curve (0–5000 pg/mL). When necessary, samples were diluted accordingly to fit within the range of the standard curve.

### MTT Cell Viability Assay

The protocol used was based on the protocol of Twentyman and Luscombe [38]. Endothelial cells were plated at 1 x 10^4^ cells/well and NT2/A cells at 1.25 x 10^4^ cells/well in a 96 well plate. After the treatment period 10 *μ*L of 5 mg/mL MTT stock solution in PBS was added to each well, and left to incubate at 37°C for 4 hours. 50 *μ*L DMSO (Sigma) was added to dissolve the crystalline product. Viability was determined by measuring the absorbance at 595 nm. The data was standardised to each control as an internal standard.

### Alamar Blue Cell Viability Assay

The assay was carried out as per the manufacturers instructions. Co-cultures were established as described above. At 48 hours after plating of the hCMVEC cells, 10 *μ*L or 100 *μ*L (1:10 volume) of alamar Blue was added to the co-culture insert and 24 well dish, respectively. Twenty four hours later 100 *μ*L of media was removed to a clean 96 well plate and the amount of fluorescence was determined using a FLUOstar Optima plate reader (excitation 544 nm, emission 590 nm). Cell free media controls were included for all experiments.

### Western Blot

Following treatments NT2/A and hCMVEC cell lystes were prepared using 1x Laemmli lysis buffer Protein concentrations in the lysate sample were measured using a commercially available assay (Bio-Rad). Prior to western blotting, aliquoted protein samples were diluted in 10x loading buffer (1M DTT + bromophenol blue (Sigma)) to achieve equal concentrations (10 *μ*g) and heated at 95°C for 5 minutes. Ten micrograms of sample were separated on a 4–12% SDS-PAGE gel (Bio-Rad) and transferred to a nitrocellulose membrane. Following blocking, membranes were incubated overnight at 4°C with rabbit anti-MCP-1 (ABM #ab9669, 1:500) or rabbit anti-NF*κ*B (Santa Cruz #sc-372 1:50). Blots were then incubated with anti-rabbit HRP-conjugated secondary antibody (Sigma #A0545. 1:16000). Bands were detected using the ECL-Plus system (Bio-Rad) and the Chemidoc MP system (Bio-Rad). Following stripping the membranes were blocked and re-probed for GAPDH (Sigma #G9545, 1:5000).

### NF*κ*B Translocation Fluorescence Assay

hCMVEC cells were treated with statins and fA*β*_1–42_ for 48 hours. Cells were then fixed with 4% PFA for 10 minutes and then washed three times with PBS. Cells were permeabilised with PBS-0.1% Tween-20 and then blocked with 4% normal goat serum diluted in PBS-T. Cells were incubated with rabbit anti NF*κ*B (Santa Cruz #sc-372 1:50) primary antibody overnight at room temperature. Following washes, goat-anti rabbit Alexa 568 (Invitrogen #A-11011, 1:500) and Hoechst (Invitrogen #H3570, 1:1000) were added for two hours at room temperature. After subsequent washes the cells were imaged at 10x magnification on an EVOS FL Auto Microscope. Translocation of NF*κ*B was observed by visualising co-localisation of Hoechst and Alexa-568 fluorescence.

### Statistical Analysis

Statistical analysis was carried out using the two-way analysis of variation (ANOVA) (Prism 6) to determine if variance was significantly differed between treatments. This was followed by a Tukeyś or Bonferroniiś multiple comparison post-hoc test. Statistically significant differences were determined where *P<0.05; **P<0.01; ***P<0.001. Data was excluded if a data point was three standard deviations from the mean as it was considered to be an outlier.

## Results

### Fibrillary A*β*_1–42_ induces pro-inflammatory cytokine release from hCMVEC and NT2/A cells, which is inhibited by statins

**Fig 1:**
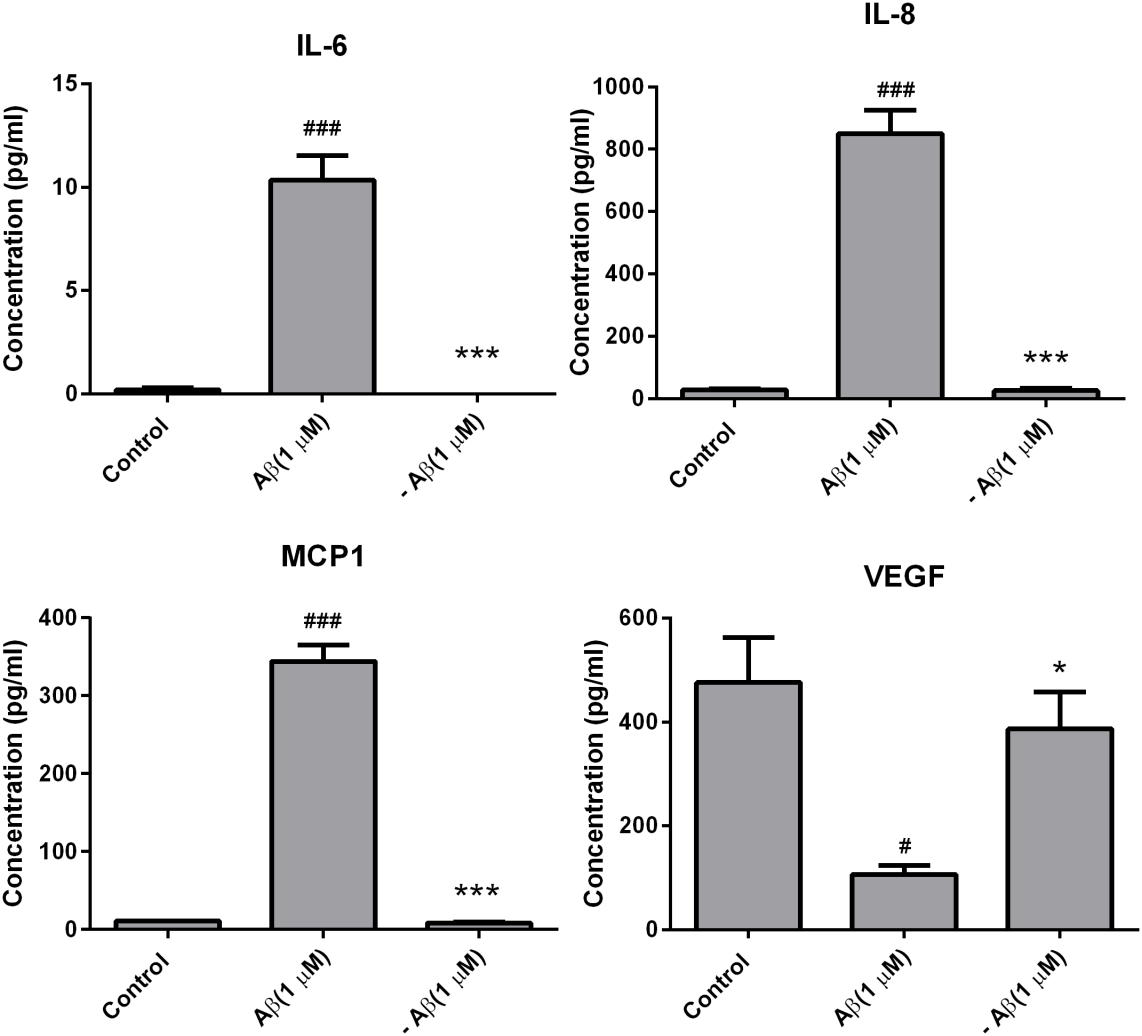
Fibrillary A*β*_1–42_-induced inflammatory cytokine production by hCMVEC cells. Each graph shows the secretion of a particular cytokine in response to stimulation by fA*β*_1–42_. Each point represents the mean ± SEM (n = 9).

**Fig 2:**
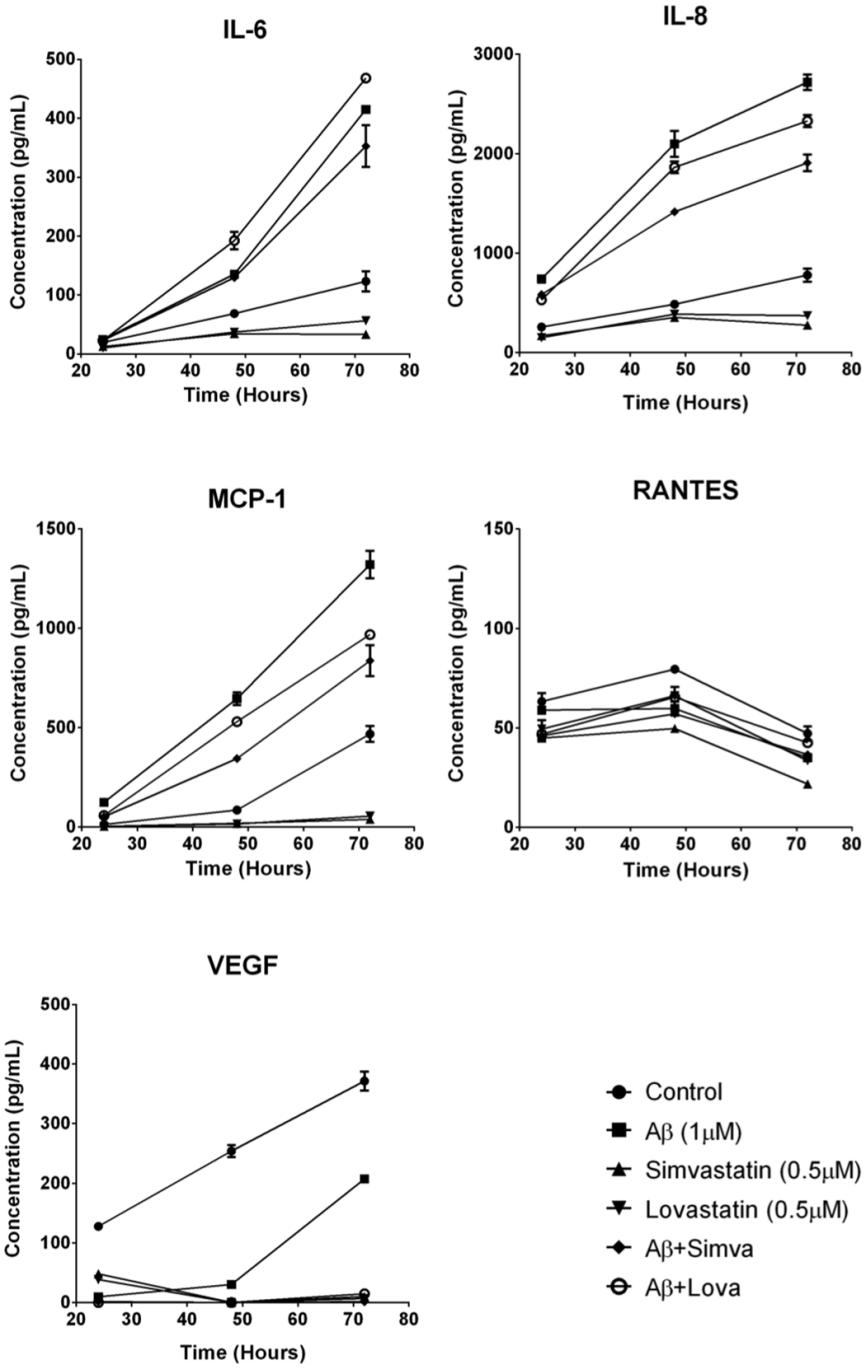
Statins reduce fA*β*_1–42_-induced inflammatory cytokine production by hCMVEC cells. Each graph shows the secretion of a particular cytokine in response to stimulation by fA*β*_1–42_ and/or statins. Each point represents the mean ± SEM (n = 9 for each 24 and 48 hour time points, n = 6 for 72 hour time point).

**Fig 3:**
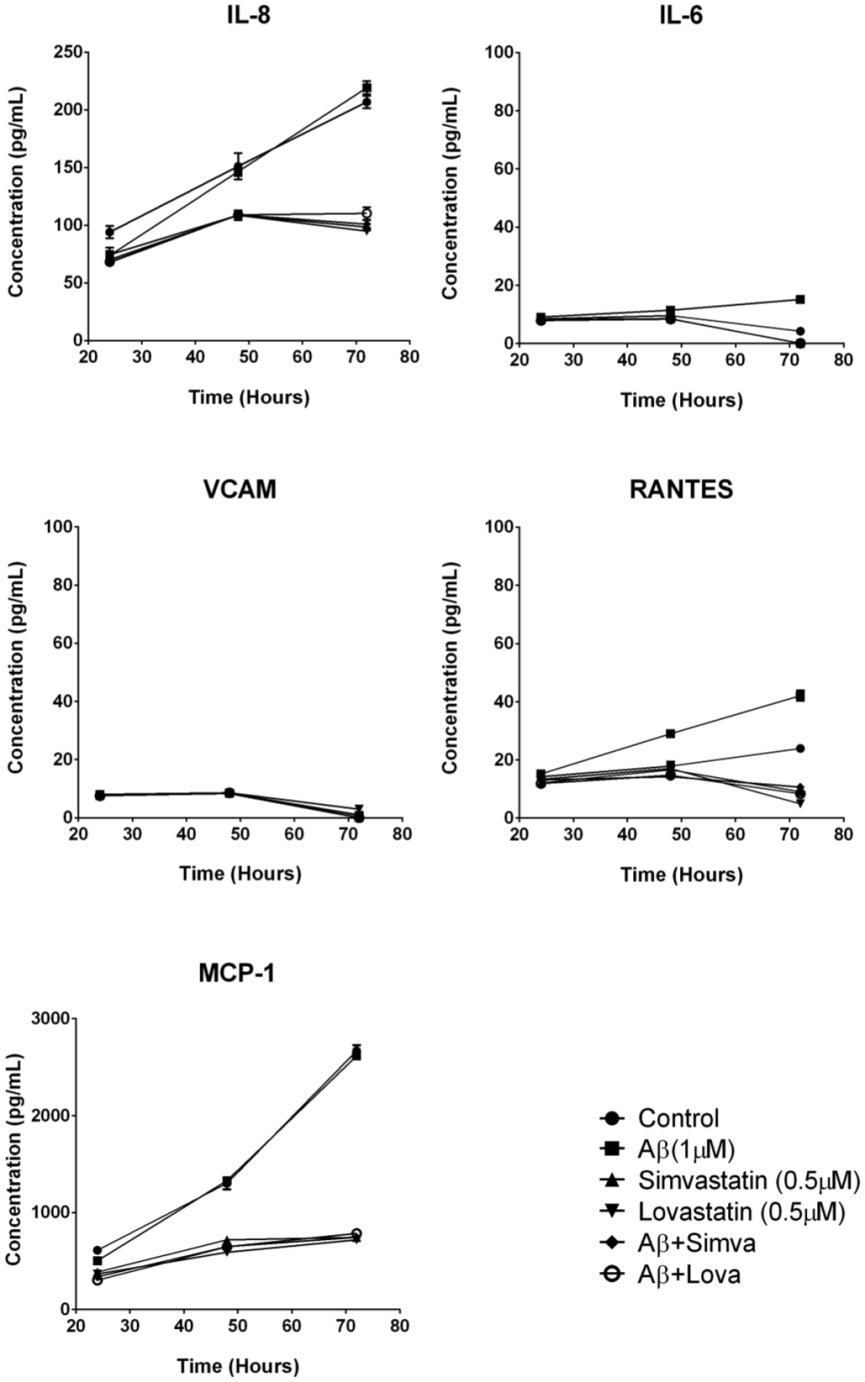
Statins reduce fA*β*_1–42_-induced inflammatory cytokine production from NT2A cells. Each graph shows the secretion of a particular cytokine in response to stimulation by fA*β*_1–42_ and/or statins. Each point represents the mean ± SEM (n = 9 for each 24 and 48 hour time points, n = 6 for 72 hour time point).

**Fig 4:**
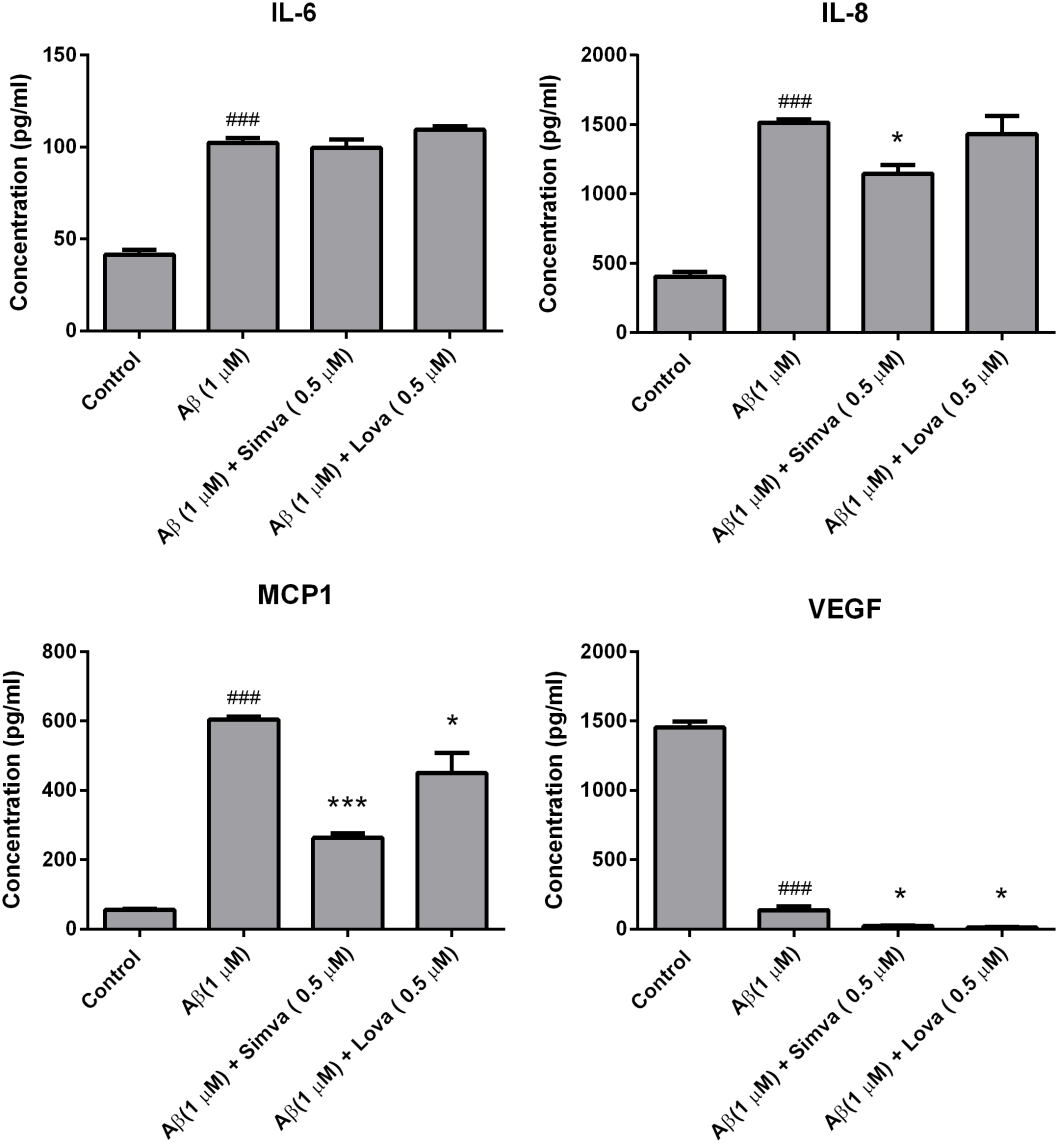
Statins inhibit fA*β*_1–42_ induced cytokine release from hCMVEC cells in a model of the BBB. A co-culture of hCMVEC and NT2/A cells were treated with 0.5 *μ*M simvastatin or lovastatin (apical side) and 1 *μ*M fA*β*_1–42_ (basolateral side) for 48 hours. Each graph shows the secretion of a particular cytokine in response to stimulation by fA*β*_1–42_ in the presence or absence of statins. Data is displayed as mean SEM (n = 9/group). (#) compares f*β*_1–42_ versus control; ###p<0.001. (*) compares statin + fA*β*_1–42_ versus statin, *p<0.05, ***p<0.001.

To determine if fA*β*_1–42_ is inflammatory to hCMVEC cells, we investigated changes in cytokine secretion following treatment of hCMVEC monocultures with 1 *μ*M fA*β*_1–42_. Based on our previous study, hCMVEC cells secret very low levels of interleukin-6 (IL-6), interleukin-8 (IL-8), monocyte chemoattractant protein (MCP-1), and vascular endothelial growth factor (VEGF) and that secretion of these was altered following activation of the cells [35]. fA*β*_1–42_ caused a significant increase in the production of the pro-inflammatory cytokines IL-6, IL-8 and MCP-1 at 48 hours post treatment (all p < 0.001) (Fig 1). Interestingly, fA*β*_1–42_ caused a dramatic reduction in VEGF concentration compared to control at 24 and 48 hours (p < 0.0001). To confirm that the effect seen was due the action of A*β*_1–42_, cytokine release was determined following application of the control fA*β*_1–42_ peptide, which has been shown to have no biological activity [39]. Treatment with fA*β*_1–42_ showed no significant increase in cytokine release compared to control cultures (Fig 1). We then tested whether statins were able to inhibit this pro-inflammatory stimulus. Simvastatin significantly reduced fA*β*_1–42_ stimulated production of IL-8, MCP-1 and IL-6 at 48 and 72 hours (all p < 0.001) (Fig 2). Lovastatin also reduced fA*β*_1–42_ stimulated production of IL-6, IL-8 and MCP-1 at 48 and 72 hours (all p < 0.0001) (Fig 2). The statin drugs did not have an effect on the fA*β*_1–42_ stimulated production RANTES, and there may have been a synergistic decrease in VEGF secretion with the combination of A*β* and statin drugs. The statin drugs on their own decreased basal cytokine secretion. Simvastatin and lovastatin (0.5 *μ*M) decreased the secretion of the cytokines; IL-6, IL-8, MCP-1, RANTES and VEGF. The effect was greatest with MCP-1 and VEGF, which were effectively reduced by more than 90% following statin treatment. In NT2/A cells, the cytokines MCP-1, IL-8, IL-6, RANTES and vascular cell adhesion protein 1 (VCAM-1) were expressed under basal conditions, consistent with our previous data [34] (Fig 3). fA*β*_1–42_ significantly increased secretion of RANTES, which was evident from 48 hours after treatment (p < 0.0001). However, there was no significant change in the secretion of MCP-1, IL-8, IL-6 or soluble VCAM-1. Similar to observations made in the hCMVEC cells, simvastatin and lovastatin had anti-inflammatory properties. Simvastatin and lovastatin completely suppressed the fA*β*_1–42_ stimulated increase of RANTES, which was the only cytokine elevated by the fA*β*_1–42_ (p < 0.0001). Simvastatin and lovastatin alone decreased the secretion of MCP-1, IL-8, and RANTES at 24, 48 and 72 hours (at 72 hours p < 0.0001) for all) but had no effect on IL-6 or VCAM-1. As described above, fA*β*_1–42_ did not cause an increase in the levels of the cytokines MCP-1, IL-8 and IL-8 produced by the NT2/A cells. While it has been reported that the NT2/A cells increase production of MCP-1, IL-8 and IL-6 in response to various pro-inflammatory cytokines, there is no previous information on how these cells respond to *β*-amyloid [34]. To confirm that the NT2/A cells used in this experiment are consistent with previous reports using these cells CBA was performed on media samples collected from cells treated with fA*β*_1–42_ as well as TNF*α*, IFN*γ* and IL-1*β*. Unlike fA*β*_1–42_, these cytokines significantly increased the levels of IL-8, IP-10, MCP-1 and IL-6 (S2 Fig). This is consistent with previous studies showing that mature human astrocytes do not release cytokine/chemokines in response to fA*β*_1–42_ [40, 41]. Western blotting was carried out to confirm the findings obtained by the CBA experiments. Consistent with the CBA results, fA*β*_1–42_ did not increase the levels of MCP-1 in NT2/A cells, statin drugs were effective in reducing the basal levels (S3 Fig). In the hCMVEC, fA*β*_1–42_ appears to increases the levels of MCP-1 while the statin drugs decreased MCP-1 in both control and fA*β*_1–42_ treated cells, consistent with the CBA data, although these results were not significant (S4 Fig). This data demonstrates that fA*β*_1–42_ is pro-inflammatory to human brain endothelial cells and astrocytes. In the above experiments fA*β*_1–42_ was added to the apical side of the endothelial cells. We then wanted to determine if basolateral application of fA*β*_1–42_ would be pro-inflammatory using a co-culture of hCMVEC and NT2/A cells as described in the methods. As seen with the monocultures, after 48 hours of treatment, basolateral application of fA*β*_1–42_ caused an increase in the release of IL-6 (p < 0.001), IL-8 (p < 0.001) and MCP (p < 0.001) in a similar fashion to that seen with apical application (Fig 4). A similar decrease in VEGF (p < 0.001) was also seen. The presence of statins inhibited the release of pro-inflammatory molecules in a similar fashion to that seen in the monocultures.

### Fibrillary A*β*_1–42_ induces NT2/A cell death, which is inhibited by statins

**Fig 5:**
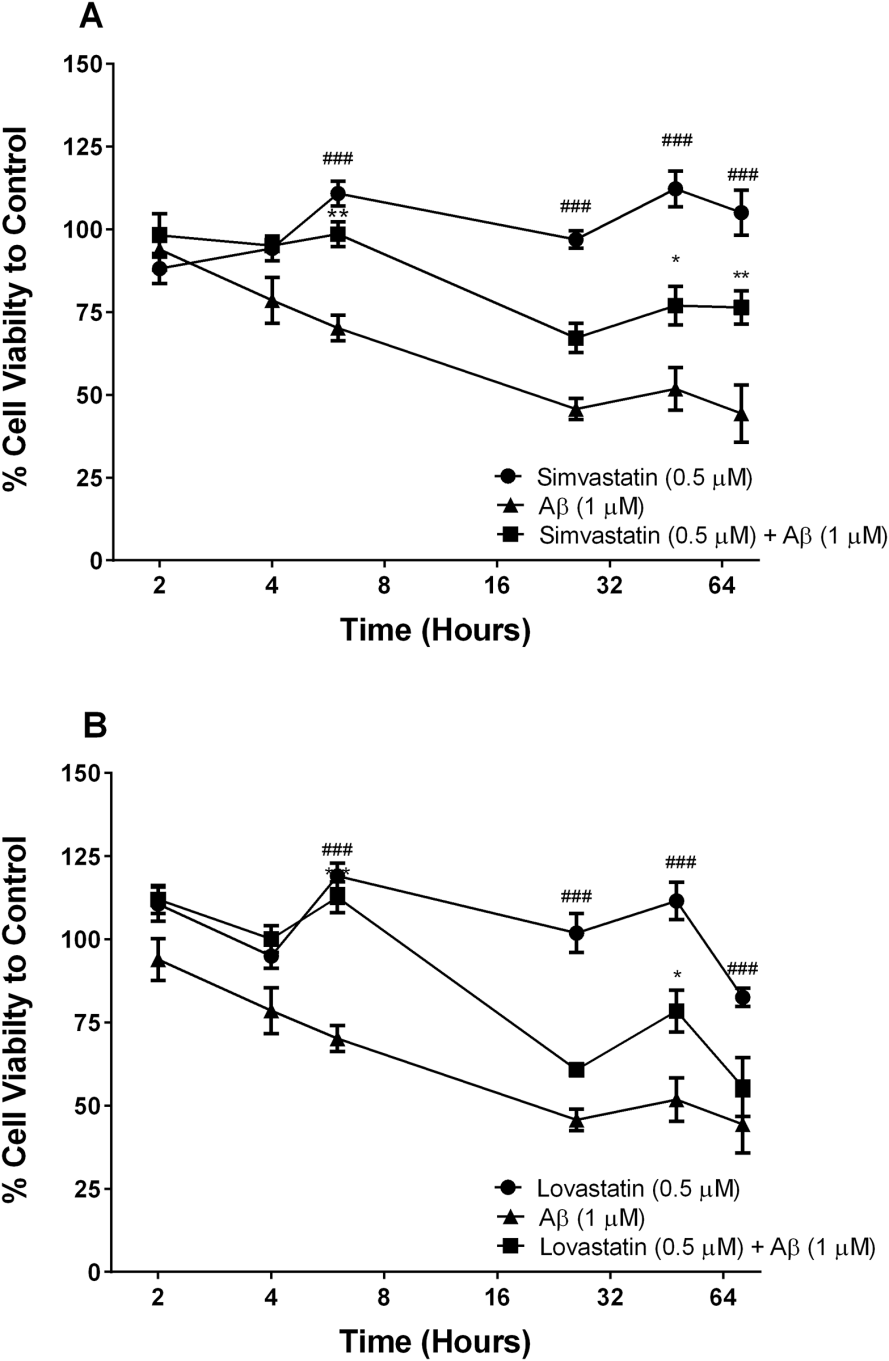
Statin drugs protect NT2-A cells against of fA*β*_1–42_ toxicity. NT2-A cells were treated with fA*β*, simvastatin ± A*β* (A), lovastatin ± A*β* (B) for up to 72 hours. Figures display the cell viability as a percentage of the vehicle control. The x-axis is a log2 scale to demonstrate early changes in viability. Data is displayed as mean ± SEM (n = 9/group). (#) compares statin versus statin + A*β* #p<0.05, ##p<0.01 ###p<0.001. (*) compares statin + A*β* versus A*β*, *p<0.05, **p<0.01, ***p<0.001.

Previous studies have shown that high concentrations of fA*β*_1–42_ are cytotoxic to astrocytes and endothelial cells of the BBB. To determine if the lower concentration used in this study was toxic, changes in cell viability of hCMVEC and NT2/A cells were analysed using MTT assay. The viability of NT2/A cells reduces rapidly (within 6 hours) following addition of fA*β*_1–42_ (Fig 5A and 5B), where there is approximately 30% reduction in cell viability. This reached a maximum of 50% after approximately 24 hours where it remained for the duration of the experiment (Fig 5A and 5B). Both simvastatin and lovastatin were shown to be significantly protective against the fA*β*_1–42_ cytotoxicity (Fig 5A and 5B).

### Fibrillary A*β*_1–42_ does not affect hCMVEC cell viability

**Fig 6:**
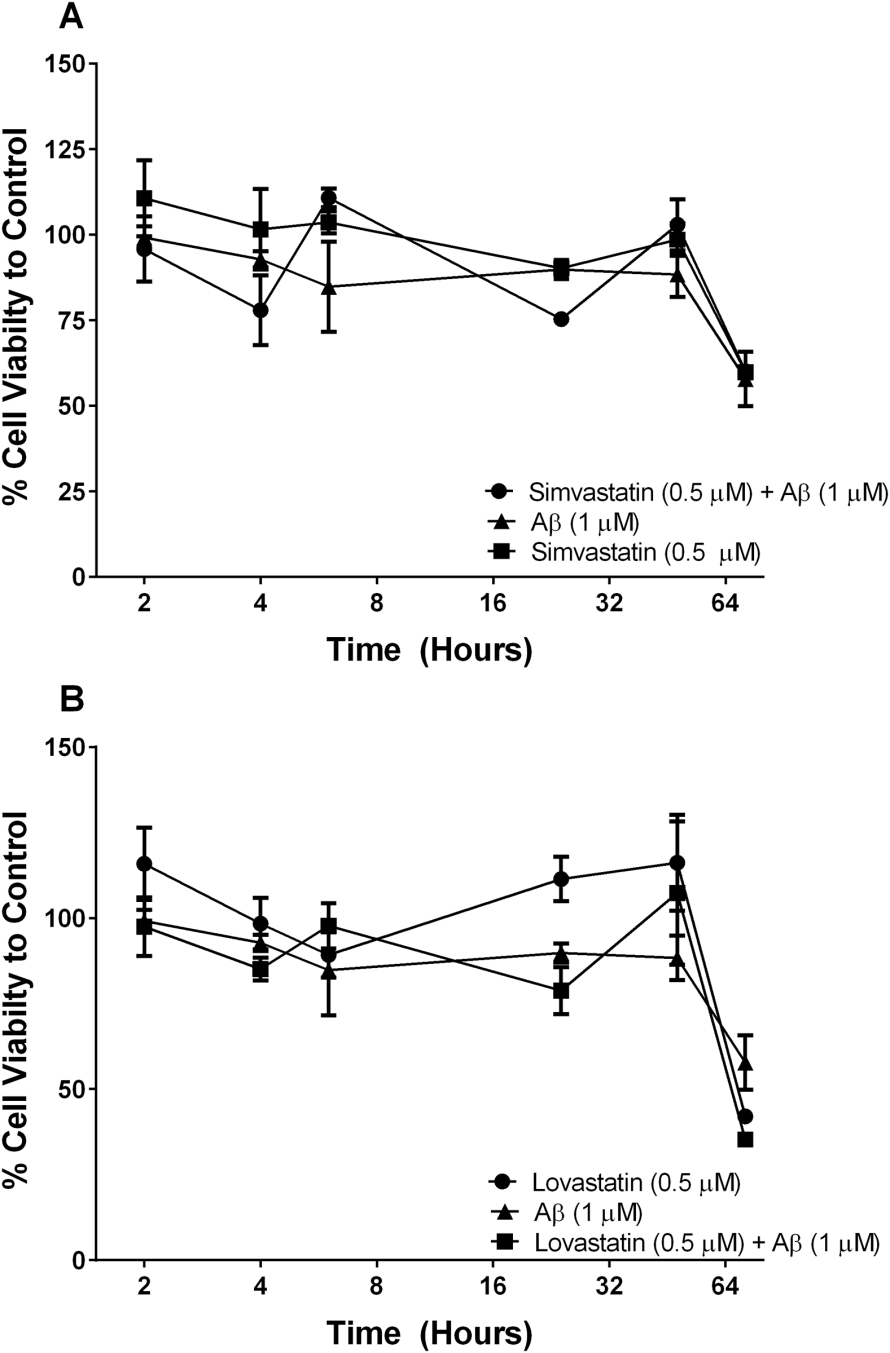
Fibrillary A*β*_1–42_ does not effect hCMVEC cell viability. hCMVEC cells were treated with A*β*, simvastatin ± A*β* (A), lovastatin ± A*β* (B) for up to 72 hours. Figures display the cell viability as a percentage of the vehicle control. The x-axis is a log2 scale to demonstrate early changes in viability. Data is displayed as mean ± SEM (n = 9/group). (#) compares statin versus statin + A*β*_1–42_ #p<0.05, ##p<0.01 ###p<0.001. (*) compares statin + A*β* versus A*β*, *p<0.05 **<0.01 ***p<0.001.

**Fig 7:**
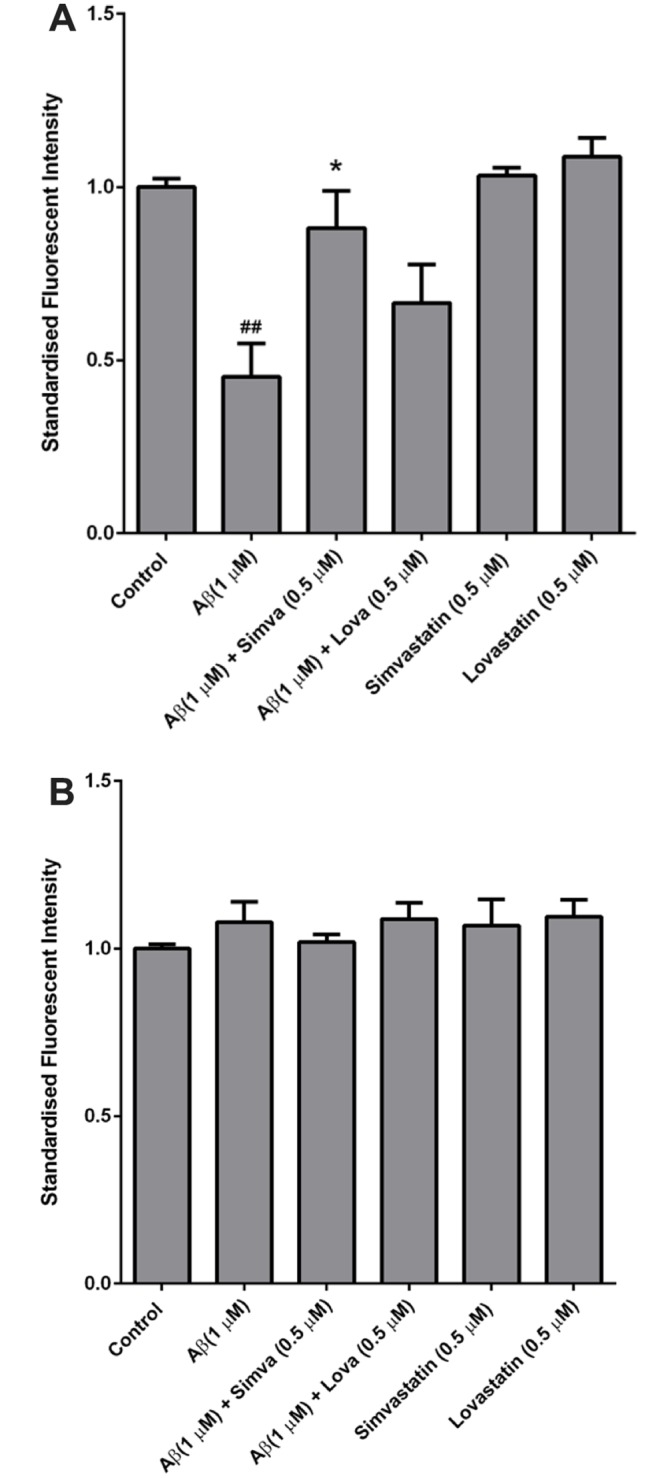
Statins inhibit fA*β*_1–42_ induced astrocyte toxicity in a model of the BBB. A co-culture of hCMVEC and NT2/A cells was treated with 0.5 *μ*M simvastatin or lovastatin (apical side) and 1 *μ*M fA*β*_1–42_ (basolateral side) for 72 hours. Figures display the cell viability relative to vehicle control. Data is displayed as mean ± SEM (n = 9/group). (#) compares fA*β*_1–42_ versus control; #p<0.05. (*) compares statin + A*β* versus statin, *p<0.05.

fA*β*_1–42_ did not have any effect on hCMVEC cell viability (Fig 6A and 6B). At 72 hours after fA*β*_1–42_ an approximately 50% reduction in cell viability was seen in all samples and this is likely due to exhaustion of nutrients in the medium in these experiments. Further experiments using the ECIS and co-cultures showed no change in resistance or TEER with apical application of fA*β*_1–42_ suggesting it is not toxic to the endothelial cells. Simvastatin or lovastatin alone did not affect the cell viability. Our data show that statins are able to protect against fA*β*_1–42_ induced cell death of NT2/A cells. To determine if exposure of the endothelial layer of the BBB to statins was able to inhibit astrocyte cell death we again used a co-culture model of hCMVECs and NT2/A cells. As demonstrated in Fig 7A, when fA*β*_1–42_ was applied to the basolateral side of the model and statins applied to the apical side, there was a marked decrease in astrocyte viability as assessed by alamar Blue (p < 0.01). Addition of simvastatin to the apical side of the co-culture significantly increased cell viability compared to fA*β*_1–42_ (p < 0.05). Lovastatin also appeared to show an increase in viability, but this was not significant. Loss of astrocyte cell viability did not have an effect on the viabilty of the endothelial cells, as determined by alamar Blue (Fig 7B).

### Basolateral but not apical application of fA*β*_1–42_ causes loss of BBB integrity

**Fig 8:**
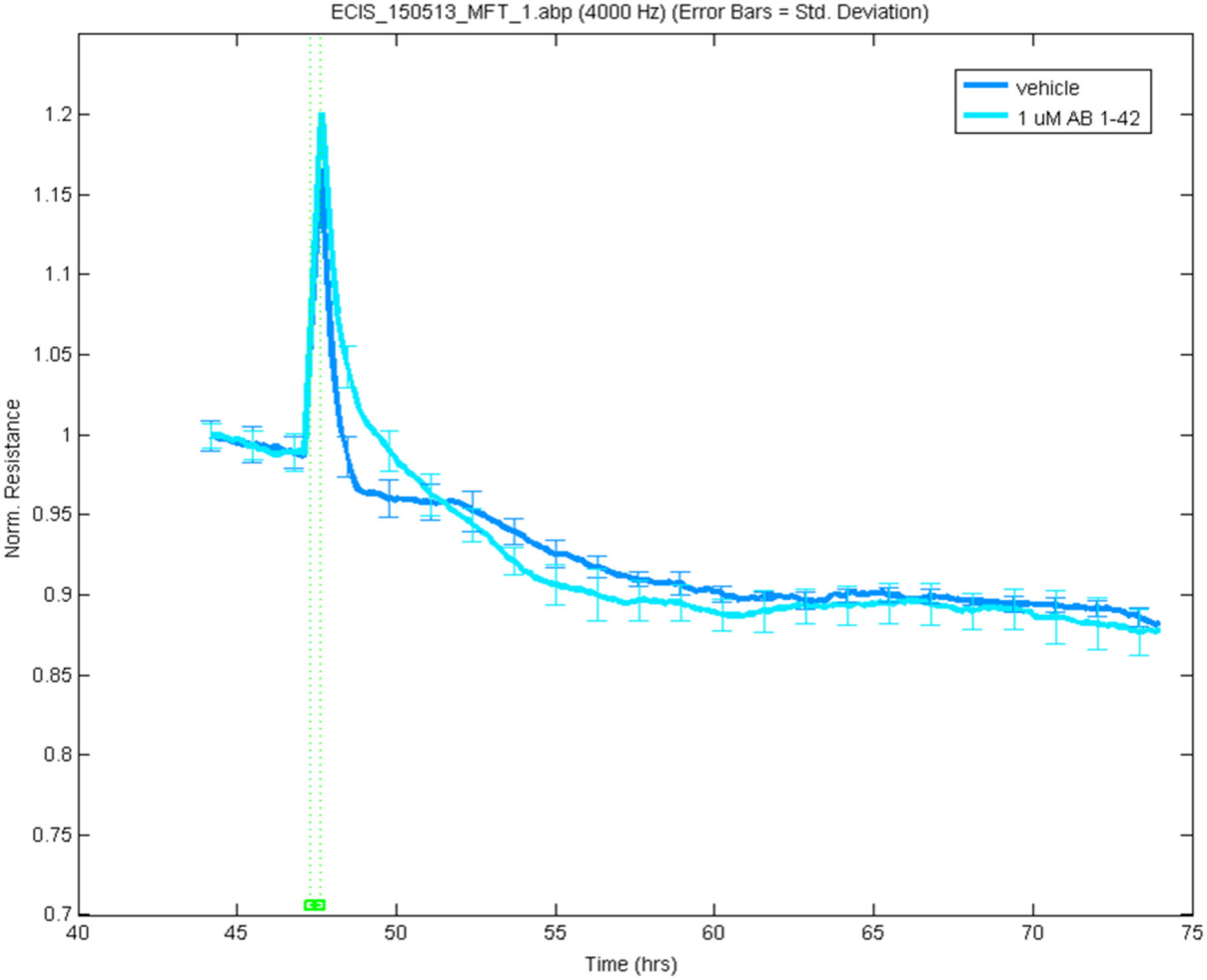
Apical addition of fA*β*_1–42_ does not effect endothelial barrier resistance. hCMVEC cells were treated with A*β*_1–42_ and barrier resistance was measured using the ECIS ZΘ at 4000 Hz. Readings were normalised at 45 hrs post plating to allow comparison of treatments. Figure is a representative image of 3 experiments, 6 wells per treatment for each experiment.

**Fig 9:**
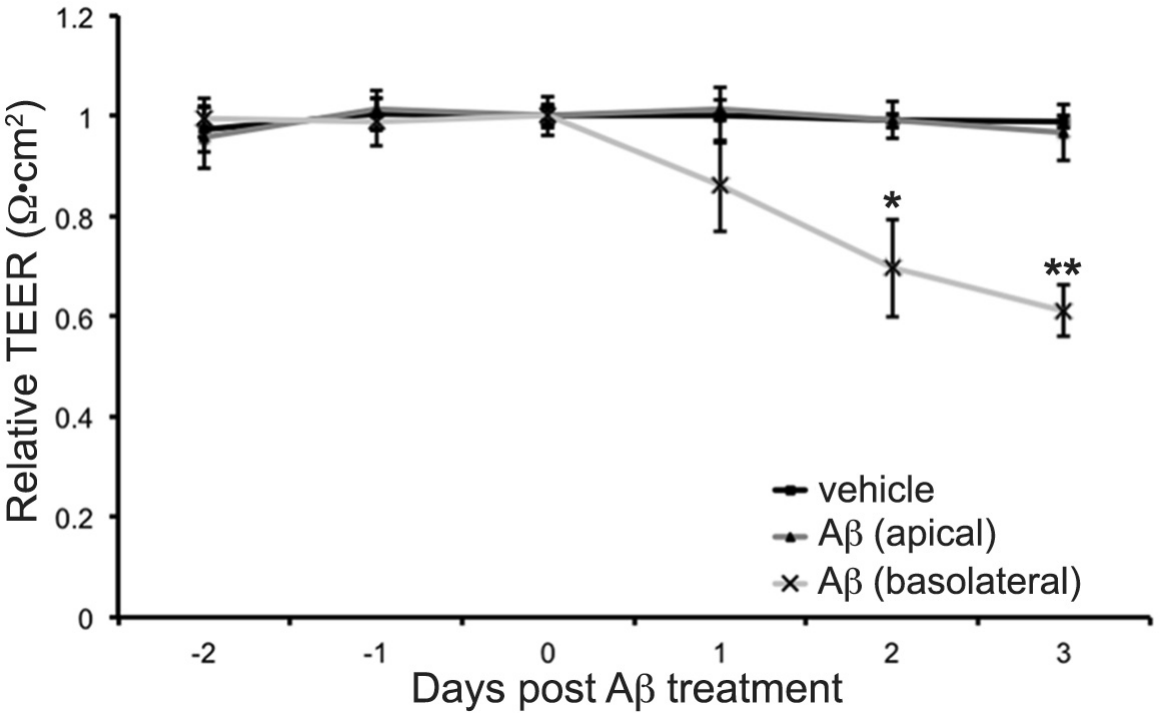
Basolateral but not apical application of fA*β*_1–42_ affects co-culture barrier integrity. Co-cultures were treated with fA*β*_1–42_ on either the apical (endothelial) or basolateral (astrocyte) side of the co-culture for 72 hours. Values are normalised to control and presented as mean ± SD (n = 3/group). *p<0.05 **<0.01 compared with vehicle.

The data above shows that 1 *μ*M fA*β*_1–42_ is not cytotoxic to hCMVEC cells. To determine if fA*β*_1–42_ can affect hCMVEC barrier integrity independent of this, ECIS ZΘ technology was used. Forty eight hours after plating, when the maximum resistance had been obtained, cells were treated with 1 *μ*M A*β*_1–42_. Fig 8 shows that fA*β*_1–42_ has no effect on hCMVEC barrier integrity. As the ECIS ZΘ is measuring only the effects of apical application of fA*β*_1–42_, we then determined the effect of basolateral addition of fA*β*_1–42_. To do this we used the co-culture model of the BBB. One micromolar fA*β*_1–42_ was added to either the apical or basolateral side of the model. As seen in Fig 8, apical application of fA*β*_1–42_ had no effect on the integrity of the barrier, a similar result to that seen with the ECIS ZΘ. However, application of fA*β*_1–42_ to the basolateral side resulted in a marked decrease in barrier integrity of approximately 40% compared to vehicle, over the 72 hours of the experiment (Fig 9).

### Statins protect the BBB against A*β*_1–42_ induced loss of integrity

**Fig 10:**
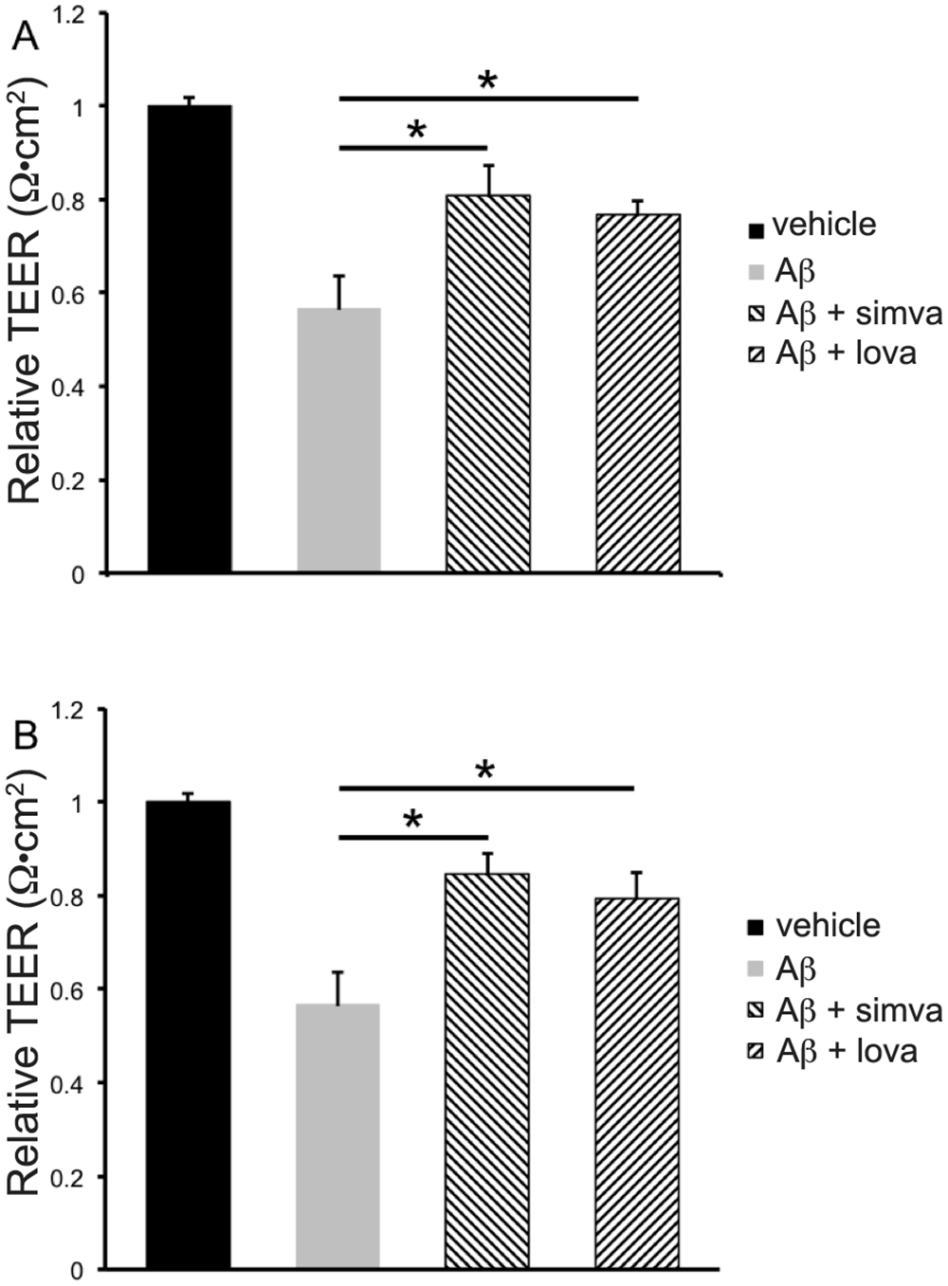
Statin drugs protect against fA*β*_1–42_ induced loss of barrier integrity. Co-cultures were treated with fA*β*_1–42_ on the basolateral (astrocyte) side of the co-culture for 72 hours in the presence of statins appled to the apical (A) or basolateral (B) side. Data is normalised TEER values 72 hours after addition of fA*β*_1–42_ statins. Data is presented as mean ± SD (n = 3/group). *p<0.05 **<0.01 compared with fA*β*_1–42_.

We then set out to determine if statins were able to protect the loss of barrier integrity caused by fA*β*_1–42_. fA*β*_1–42_ was applied to the basolateral side of the culture in conjunction with 0.5 *μ*M simvastatin or lovastatin applied to either the apical or basolateral side of the co-culture. Fig 10 shows that both simvastatin and lovastatin are protective of barrier integrity when applied to either the apical (Fig 10A) or basolateral (Fig 10B) side of the co-culture.

### Fibrillary A*β*_1–42_ does not induce NF*κ*B expression but statins reduce the levels of NF*κ*B

**Fig 11:**
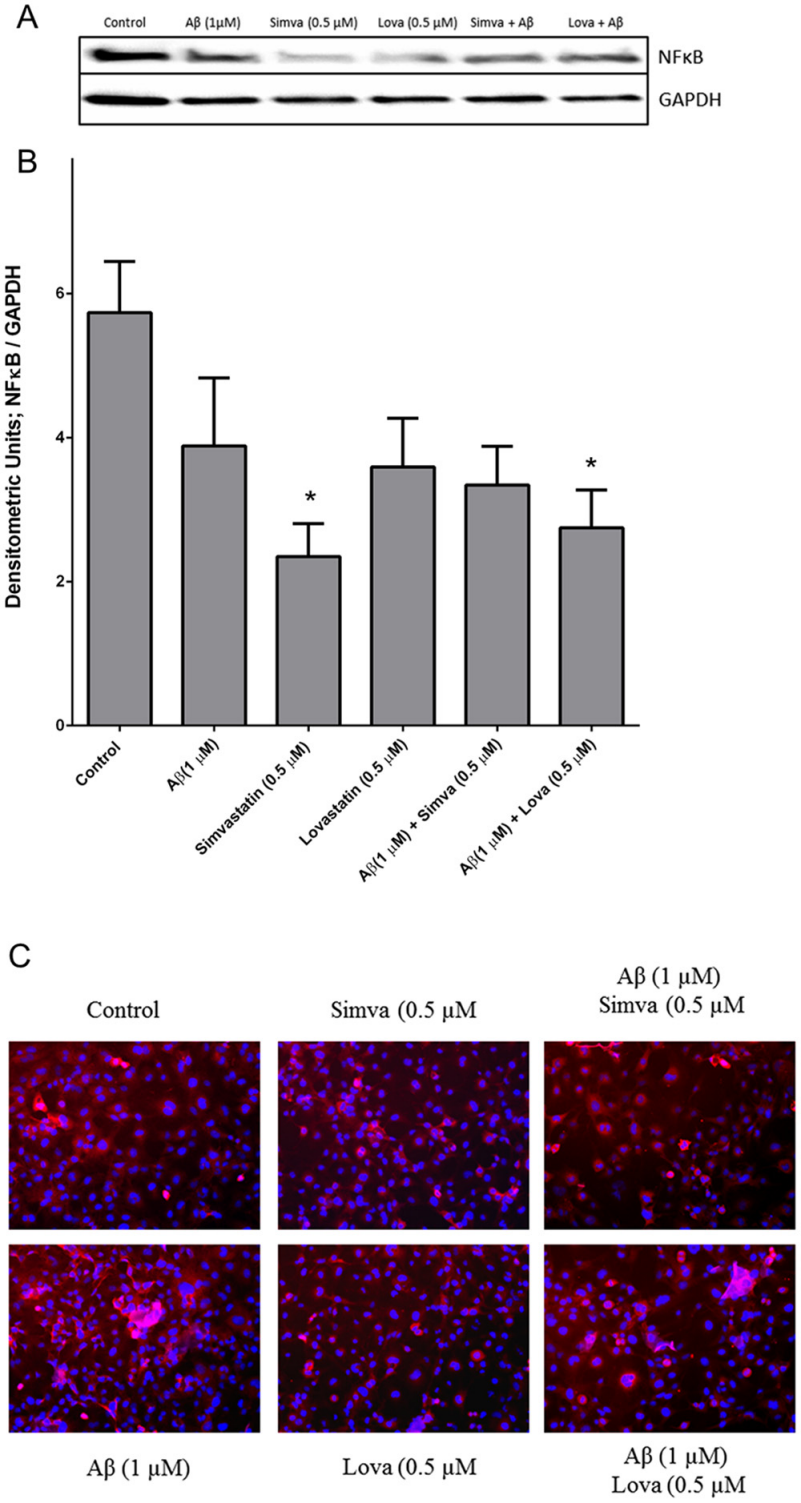
Fibrillary A*β*_1–42_ does not cause activation of the NF*κ*B pathway. NF*κ*B p65 subunit is not translocated into the nucleus in the presence of fA*β*_1–42_ (A). Western blotting showing relative NF*κ*B protein levels following treatment with fA*β*_1–42_ and statins (B and C). Data is presented as mean ± SD (n = 5). *p<0.05 compared with control.

The NF*κ*B pathway has been shown to be involved in the pathological effects of A*β* [42–44]. It has also been reported that the anti-inflammatory effects of statins work at least in part through inhibition of the NF*κ*B pathway [45–47]. We therefore carried immunohistochemistry and Western blotting to determine if the protective effects of statins where through inhibition of the NF*κ*B pathway. Analysis of NF*κ*B p65 translocation to the nucleus by immunohistochemistry showed that fA*β*_1–42_ did not cause translocation (Fig 11A). When the levels of NF*κ*B protein were measured by Western blotting, fA*β*_1–42_ did not lead to an increase in protein levels (Fig 11B and 11C). Interestingly, simvastatin alone was able to reduce NF*κ*B protein levels as was lovastatin when added in the presence of fA*β*_1–42_.

## Discussion

The aims of this study were to investigate the effect of statin drugs on fibrillary *β*-amyloid1-42 (fA*β*_1–42_) induced changes in a model of the human BBB using analysis of cytokine production, cell toxicity and barrier integrity. In AD, a number of different forms of A*β* peptides are present; monomeric, oligomeric and fibrillary and all have been reported to have different effects. It is important to understand the different effects each form can have at the BBB. Monomeric A*β* has been shown to affect endothelial cell tight junctions, barrier integrity and viability [11–14]. Oligomeric A*β* is believed to be the most inflammatory form [13, 14, 48–51] with studies that have compared the forms have showing oligomeric to increase permeability to a greater extent than monomeric or fibrillary A*β*. In a study using mouse derived bEND2 endothelial cells, toxicity was seen following treatment with the monomer, oligomer and fibrillary forms of A*β*. When barrier function was studied, the oligomeric form of A*β*_1–42_ had the greatest effect on permeability [14]. A study in the human brain endothelial cell line hCMEC/D3 demonstrated that treatment of cells with the monomeric and oligomeric forms of A*β*_1–42_ in combination led to toxicity but no change in viability was seen when they were applied separately [12]. The studies above describe both apical and basolateral treatment with A*β* forms and show both applications to be capable of toxicity to endothelial cells. Previous reports have shown that fA*β*_1–42_ causes cell death in endothelial cells of the blood brain barrier, using both animal and cell culture models. However, these reports use high (10 *μ*M) concentrations of fA*β* [15, 32, 52, 53]. We provide evidence that fA*β*_1–42_ at a lower concentration (1 *μ*M) can induce cytokine release from human brain endothelial cells while not causing cytotoxicity or loss of barrier integrity. While soluble A*β* is thought to be the main A*β*_1–42_ species that is involved in cell toxicity in AD, fibrillary A*β*_1–42_ also plays an important role. Cerebral amyloid angiopathy (CAA) is often seen in AD and is caused by the deposition of A*β*_1–42_ aggregates in brain vasculature. This leads to disruption of blood vessels and disturbed cerebral blood flow [31]. Our data suggests that the presence of fibrillary A*β*_1–42_ at blood vessels may have an inflammatory effect in the absence of endothelial cell death or loss of endothelial barrier integrity. The pro-inflammatory cytokines IL-6, IL-8 and MCP-1 were increased in response to fA*β*_1–42_ and this response is similar to that of primary cerebral endothelial cell lines [54, 55]. Fibrillary A*β*_1–42_ causes a reduction in VEGF. VEGF is known to have neurotrophic and neuroprotective effects [56]. It has previously been reported that VEGF has a strong affinity (KD ~50 pM) for A*β* aggregation and so it is sequested by the amyloid plaques, which would explain what we observed [56]. Therefore this could imply that A*β* may prevent the beneficial effects that are associated with VEGF. The secretion of cytokines/chemokines was similar regardless of whether fA*β*_1–42_ was added to the apical surface of the endothelial cells or the basolateral side of a co-culture system including astrocytes. This suggests that the cytokine release is a direct effect of fA*β*_1–42_ on the endothelial cells as opposed to a downstream effect of its action on astrocytes. The clinical outcome of this inflammation is likely to result in immune cell infiltration that will result in neuroinflammation in the brain and lead to greater damage. There are examples of the negative impact of IL-6 and IL-8 on the permeability of the BBB through down-regulation of tight junctions [57]. MCP-1 is a chemokine that recruits monocytes, memory T cells, and dendritic cells increasing BBB permeability and immune infiltration [58]. AD patients have an increased presence of monocytes/macrophages in their vessel walls [59], which can be explained by increased release of chemokines from endothelial cells. We show that fA*β*_1–42_ leads to NT2/A astrocyte cell death, in line with other studies showing A*β*_1–42_ induced death and dysfunction of astrocytes [60–62]. fA*β* has been shown to reduce glut-1 transporter and cause retraction of astrocytic end feet and cell swelling, and cause inflammation in rat astrocyte cultures [16, 32]. In our study, the cytotoxic effect of the fA*β*_1–42_ on the astrocytes was very rapid, being evident within 6 hours. This suggests that changes in astrocyte function may occur very early after the deposition of A*β*_1–42_ at the blood brain barrier. In the NT2/A cells fA*β*_1–42_ increased the secretion of the chemokine RANTES but not the other cytokines. Previous data has reported that the NT2/A cells consistently respond to pro-inflammatory molecules, such as TNF-*α*, INF-*γ* and IL-1*β* [34]. We tested the responses of our NT2/A cells to these pro-inflammatory cytokines and found they increased IL-6, IL-8, IP-10 and MCP-1 in magnitudes greater than the control. Therefore, we are satisfied that the NT2/A cells are capable of cytokine secretion as has been shown previously [34]. The cytokine response of primary human adult astrocytes has not been studied in great detail. Recently it was shown that both primary adult human astrocytes and primary human fetal astrocytes did not produce MCP-1 in response to A*β*_1–42_ at concentrations up to 20 *μ*M, in fact there were significant reductions in MCP-1 release [41]. The cell viability of these cells however was negatively affected by A*β*_1–42_, consistent with what was observed in this present study. We demonstrated that fA*β*_1–42_ causes a loss of barrier integrity when applied to the basolateral but not the apical side of our BBB model containing both endothelial cells and astrocytes. This is similar to a study looking at the effect of tau on a model of the BBB. In this study, truncated tau did not cause a reduction in TEER or induce toxicity when applied apically to primary rat endothelial cells but when applied to the lower chamber of a BBB co-culture of endothelial cells, astrocytes and microglia there was a decrease in TEER and increased permeability to mannitol. This suggests the effect of aggregated A*β* at the blood vessels may act in a similar fashion to tau as opposed to soluble A*β*, where apical addition has been shown to cause cell death. While reports have shown cytotoxic effects of fA*β* on endothelial cells, these have used higher concentrations (10–20 *μ*M) than used in this study and were not carried out using co-culture systems including other cell types [15, 52]. Statin drugs protected against fA*β*_1–42_ induced cytokine release from brain microvascular endothelial cells, prevented cytotoxicity and the secretion of pro-inflammatory cytokines from astrocytes and inhibited BBB barrier compromise in our model and collectively, this would contribute towards the protection of the BBB from fA*β*_1–42_ induced barrier compromise. Simvastatin and lovastatin were effective in reducing both the basal secretion and the fA*β*_1–42_ stimulated secretion of pro-inflammatory cytokines in hCMVEC cells. Therefore statins would inhibit the negative effects of cytokines on barrier integrity and immune cells infiltration as described above. The decrease of basal cytokine secretion suggests statins may be able to protect endothelial cells from inflammatory insults by down-regulating the cells ability to initiate an inflammatory response. Statins had a cytoprotective effect on the astrocytes. This effect was seen when the statins were applied on either the apical or basolateral side of the co-culture. Due to their lipophilicity, statins have been shown to readily cross the BBB [63] and so are possibly having a protective effect directly on the astrocytes. However, the statins also had an effect on cytokine release from the endothelial cells which could at least in part explain their action. It has been shown previously that statins can reduce cytokine release from human brain endothelial cells following pro-inflammatory stimulation thereby partially preventing BBB breakdown [64]. Interestingly, the statins alone had an anti-inflammatory effect on the astrocytes through reductions in basal cytokine levels. MCP-1, IL-8 and RANTES were all significantly decreased with statin treatments. Statins have previously been reported to maintain the integrity of the BBB, for example, in response to intracerebral haemorrhage [21], or in vitro/in vivo cholesterol disruption [18]. Therefore, statins may down regulate the release of cytokines from astrocytes, protecting them against pro-inflammatory insults. A report using an endothelial cell line-astrocyte cell line co-culture model of the human BBB showed that statins are protective against A*β* induced expression of proinflammatory molecules [30]. However, in this study the authors did not measure changes in TEER or permeability or determine changes in endothelial or astrocyte viability. This study also did not look at the effects of fA*β*, so our study is the first to look at the effects of statins on fA*β* induced changes at the BBB. Our data suggests that this reduction in cytokine release is in fact protective of the human BBB. One of the key mechanisms by which fA*β* is known to be toxic is through the production of reactive oxygen species (ROS) [65–67] One of the key regulators of this process is the transcription factor NF*κ*B which is activated by the presence of fA*β* and ROS and is able to upregulate pro-oxidant genes, increase cytokine release and other inflammatory and immune signalling [68]. Statins have been shown to down-regulate the activity of NF*κ*B [45–47] so we wanted to determine if this was the mechanism by which statins inhibited the fA*β*_1–42_ effects. Interestingly, fA*β*_1–42_ did not cause translocation of the NF*κ*B p65 subunit to the nucleus or cause an increase in the level of NF*κ*B protein. This differs from other studies showing soluble A*β* acts via this pathway, and suggests fA*β* acts independently of NF*κ*B and shows statins must be inhibiting the effects of fA*β* via a different mechanism. The presence of statins was able to reduce the protein levels of NF*κ*B below those seen in control cells, both alone (in the case of simvastatin) and in the presence of fA*β*_1–42_ (in the case of lovastatin). It is unclear if this is a mechanism by which the statins are being protective but it is possible that by reducing the levels of NF*κ*B in the cells that this protects them from a future insult.

## Conclusion

In this study we have shown that stimulation of a human BBB by fA*β* is able to stimulate cytokine release from endothelial cells, independent of changes in barrier integrity or cell toxicity and that the presence of fA*β* on the basolateral but not apical side of a blood brain barrier causes loss of barrier integrity and cytotoxicity to astrocytes. This action of fA*β* is different from the soluble forms of A*β*, suggesting a different mechanism and our data suggests that fA*β* does not act via NF*κ*B, which is in contrast to the soluble forms of A*β*. Understanding how aggregated A*β* is able to affect the blood brain barrier is an important step in understanding and developing treatments for conditions such as is capillary amyloid angiopathy. Statins are protective of human blood brain barrier integrity in the presence of fA*β*_1–42_ and this is at least in part due to cytoprotective effects on astrocytes and reducing cytokine release from astrocytes and endothelial cells suggesting a possible mechanism for the protection in Alzheimer’s disease.

## Supporting Information

S1 Fig Image showing presence of aggregated A*β*_1–42_. Image of A*β*_1–42_ solution showing presence of aggregated fA*β*_1–42_ following incubation at 37°C for 5 days.
(TIF)

S2 Fig Cytokine release from NT2/A cells following stimulation by TNF*α*, IFN*γ* and IL-1*β*. Cytokine analysis confirming NT2/A cells are able to release cytokines in response to inflammatory stimulation. Data is presented as mean ± SD, n = 3. *p<0.05, ***p<0.001 compared with control.
(TIF)

S3 Fig Effects of fA*β*_1–42_ and statins on MCP-1 levels in NT2/A cells. fA*β*_1–42_ does not lead to an increase in MCP-1 protein levels in NT2/A cells, but statins alone reduce the levels of MCP-1. (A) Representative western blot, (B) Quantification of MCP-1/GAPDH ratio; Data is presented as mean ± SD, n = 3 experiments.
(TIF)

S4 Fig Effects of fA*β*_1–42_ and statins on MCP-1 levels in hCMVEC cells. fA*β*_1–42_ causes an increase in MCP-1 protein levels in hCMVEC cells, which is inhibited by statins. (A) Representative western blot, (B) Quantification of MCP-1/GAPDH ratio; Data is presented as mean ± SD, n = 3 experiments.
(TIF)

S1 Data Spreadsheet of CBA data for cytokine release from NT2/A cells. (XLS)

S2 Data Spreadsheet of CBA data for cytokine release from hCMVEC cells. (XLS)

S3 Data Spreadsheet of CBA data for cytokine release from co-cultures. (XLS)

S4 Data Spreadsheet of MTT data for NT2/A cell death. (XLSX)

S5 Data Spreadsheet of MTT data for hCMVEC cell death. (XLSX)

S6 Data Spreadsheet of alamarBlue data for co-culture cell death. (XLSX)

S7 Data Spreadsheet of data from analysis of barrier integrity. (XLSX)

S8 Data Western blots of NF*κ*B protein levels. (ZIP)

S9 Data Images of NF*κ*B localisation. (ZIP)
