# Supplementary figures and images for: Statins Inhibit Fibrillary β-Amyloid Induced Inflammation in a Model of the Human Blood Brain Barrier
Source: PLoS One. 2016 Jun 16;11(6):e0157483. doi: 10.1371/journal.pone.0157483 (PMC4911157; doi:10.1371/journal.pone.0157483)

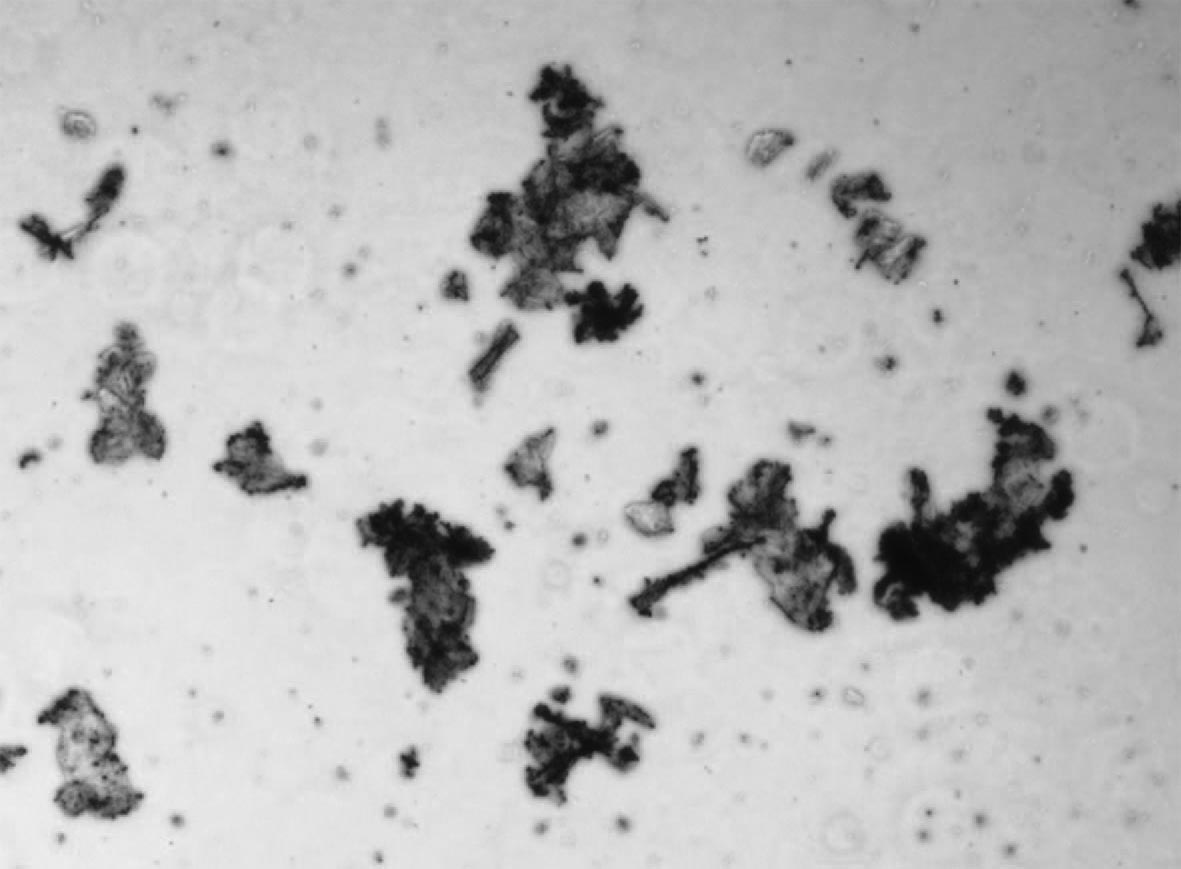

Supplement: S1 Fig — Image of Aβ1–42 solution showing presence of aggregated fAβ1–42 following incubation at 37°C for 5 days. (TIF) [file pone.0157483.s001.tif]

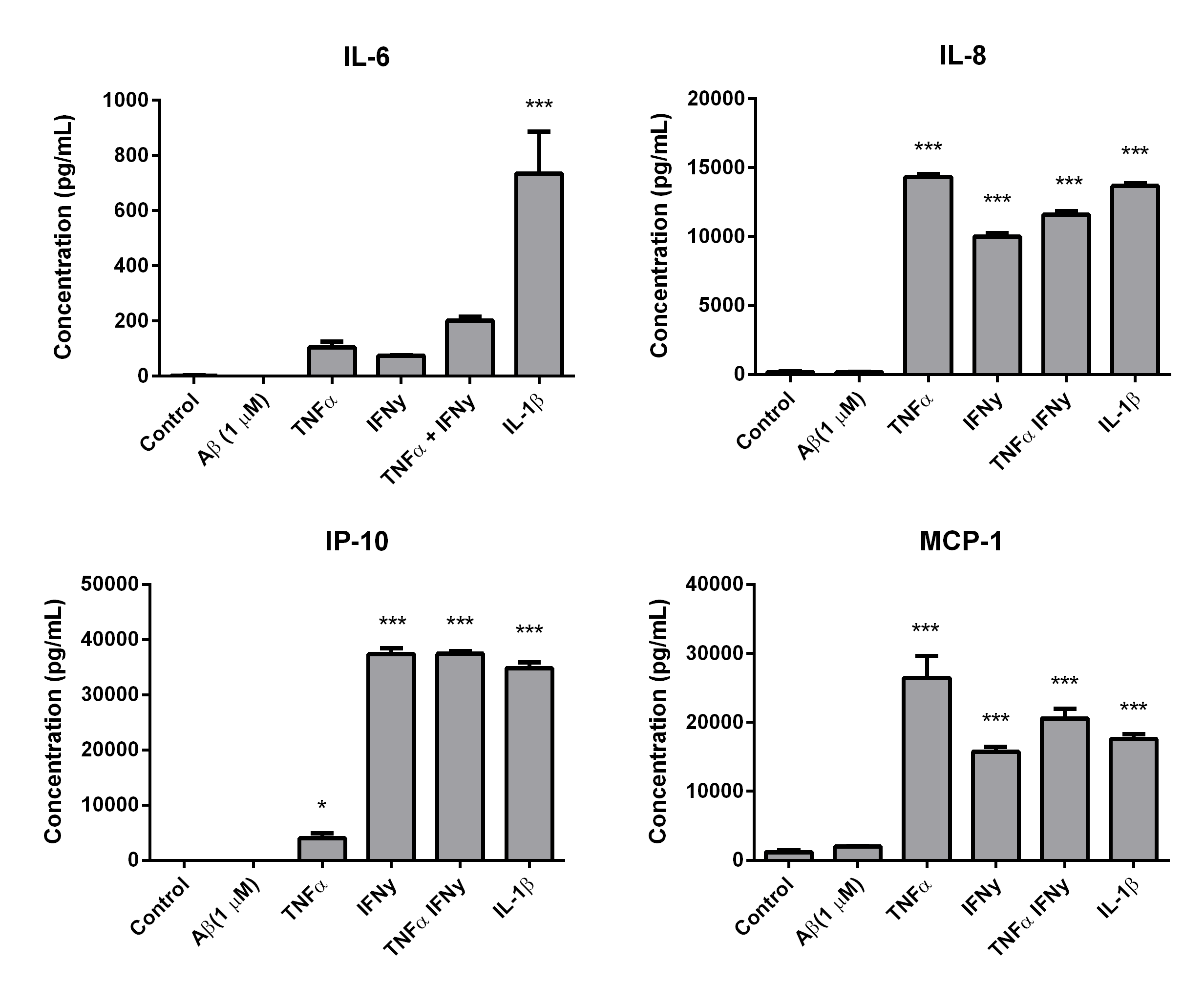

Supplement: S2 Fig — Cytokine analysis confirming NT2/A cells are able to release cytokines in response to inflammatory stimulation. Data is presented as mean ± SD, n = 3. *p<0.05, ***p<0.001 compared with control. (TIF) [file pone.0157483.s002.tif]

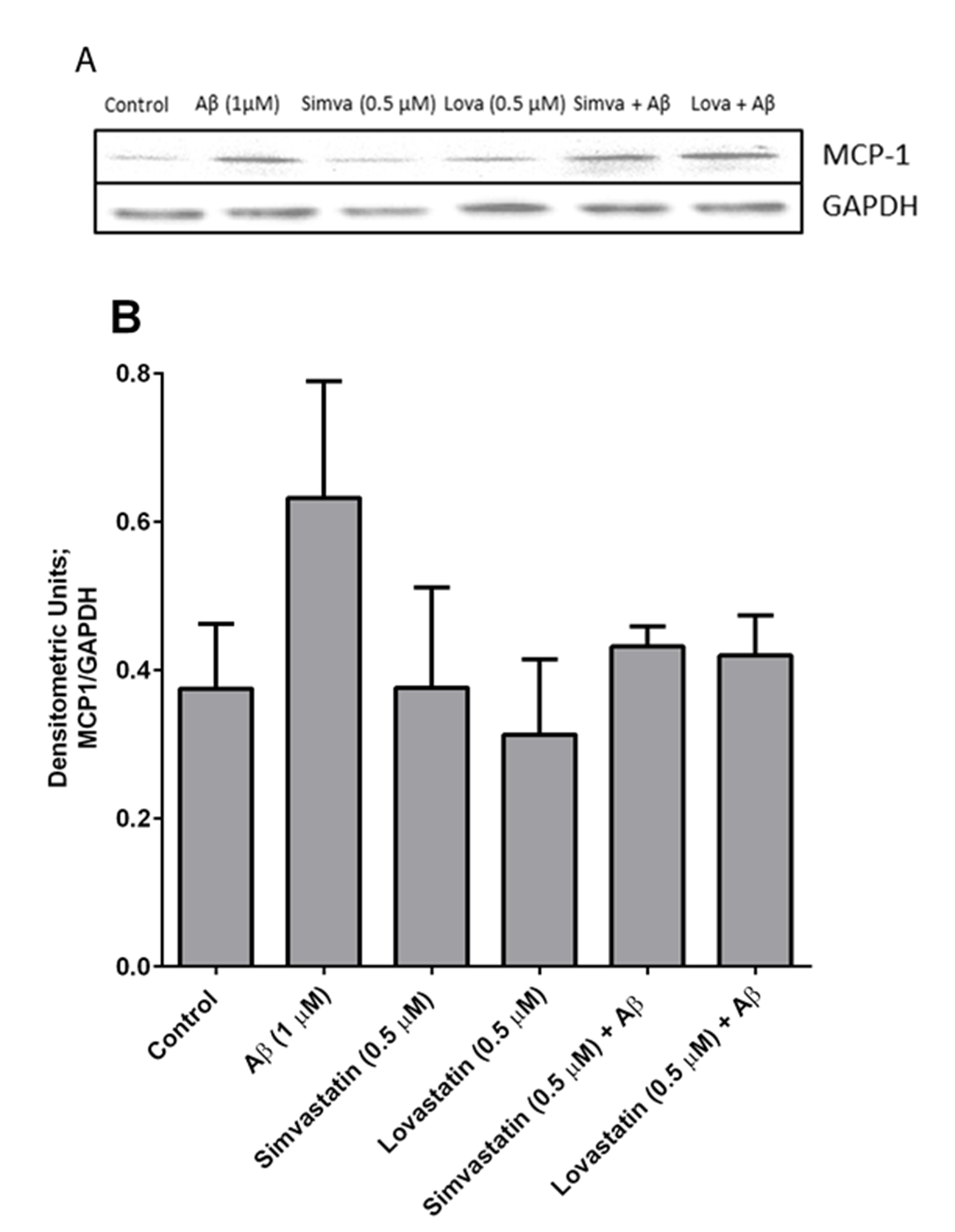

Supplement: S3 Fig — fAβ1–42 does not lead to an increase in MCP-1 protein levels in NT2/A cells, but statins alone reduce the levels of MCP-1. (A) Representative western blot, (B) Quantification of MCP-1/GAPDH ratio; Data is presented as mean ± SD, n = 3 experiments. (TIF) [file pone.0157483.s003.tif]

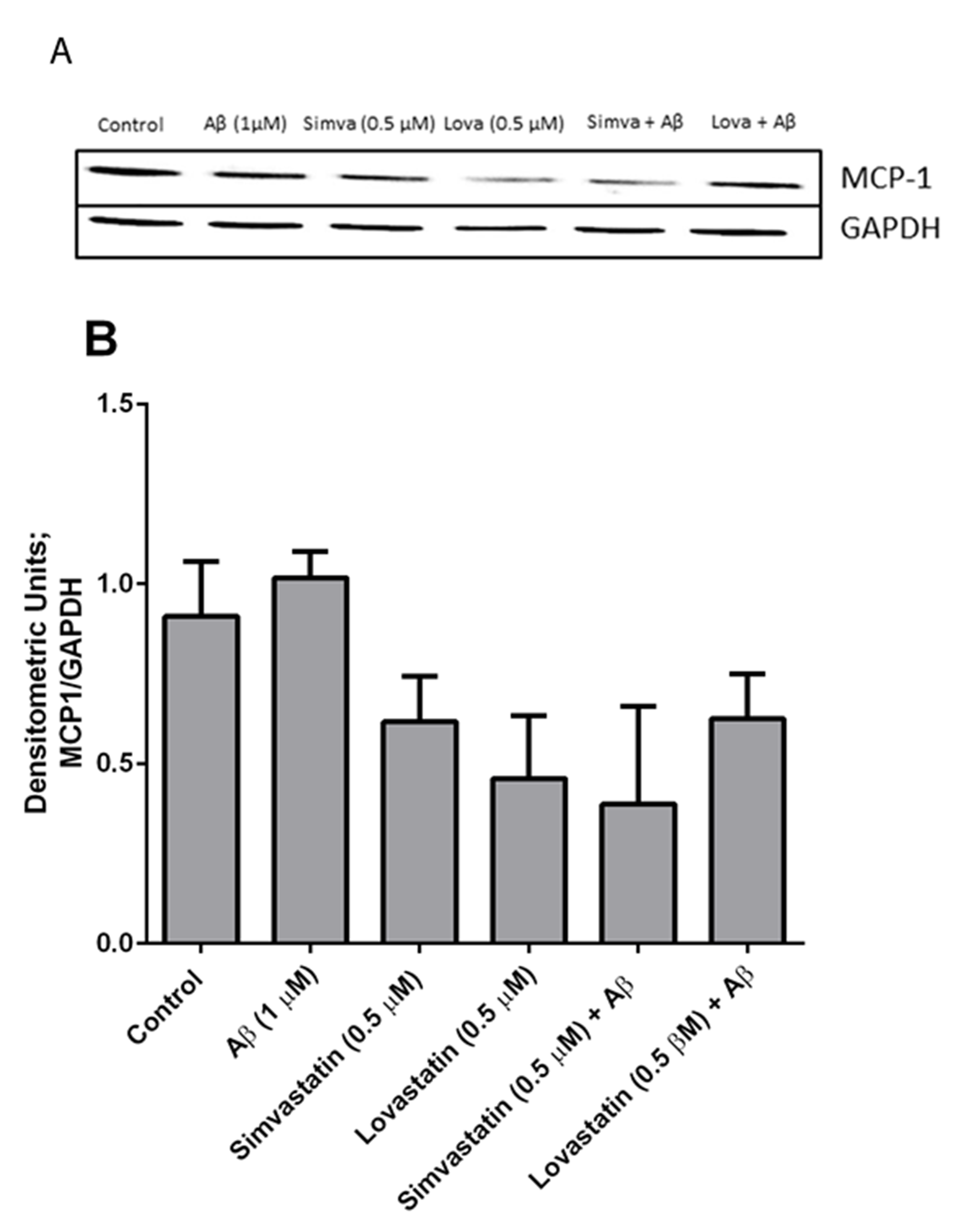

Supplement: S4 Fig — fAβ1–42 causes an increase in MCP-1 protein levels in hCMVEC cells, which is inhibited by statins. (A) Representative western blot, (B) Quantification of MCP-1/GAPDH ratio; Data is presented as mean ± SD, n = 3 experiments. (TIF) [file pone.0157483.s004.tif]

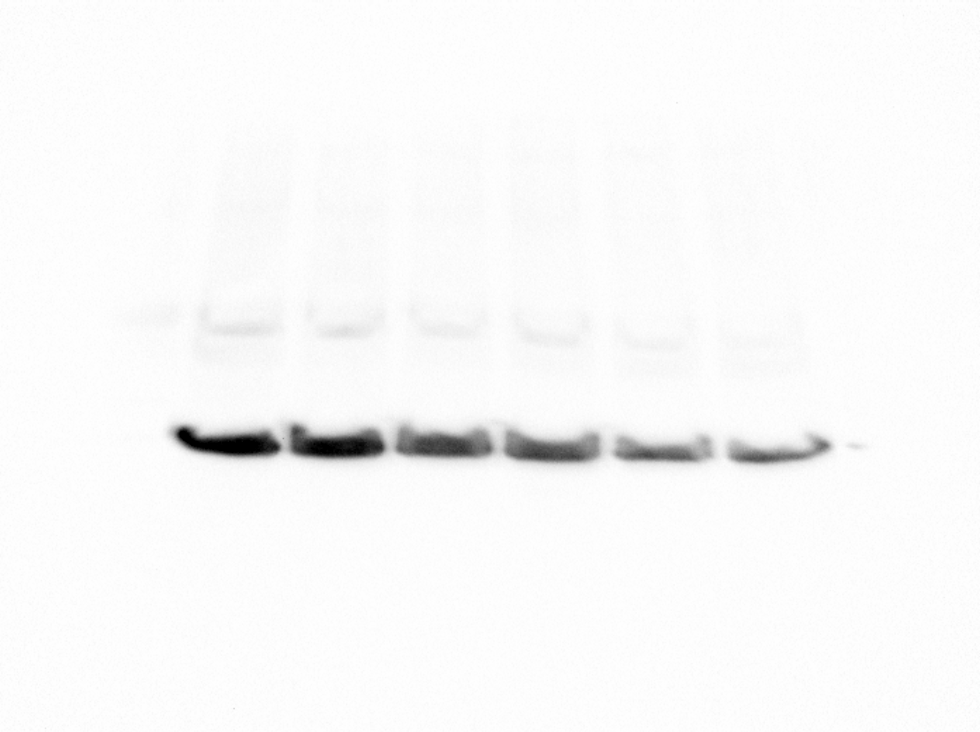

Supplement: S8 Data — (ZIP) [file pone.0157483.s012.zip › NFKB Blots/GAPDH/1..tif]

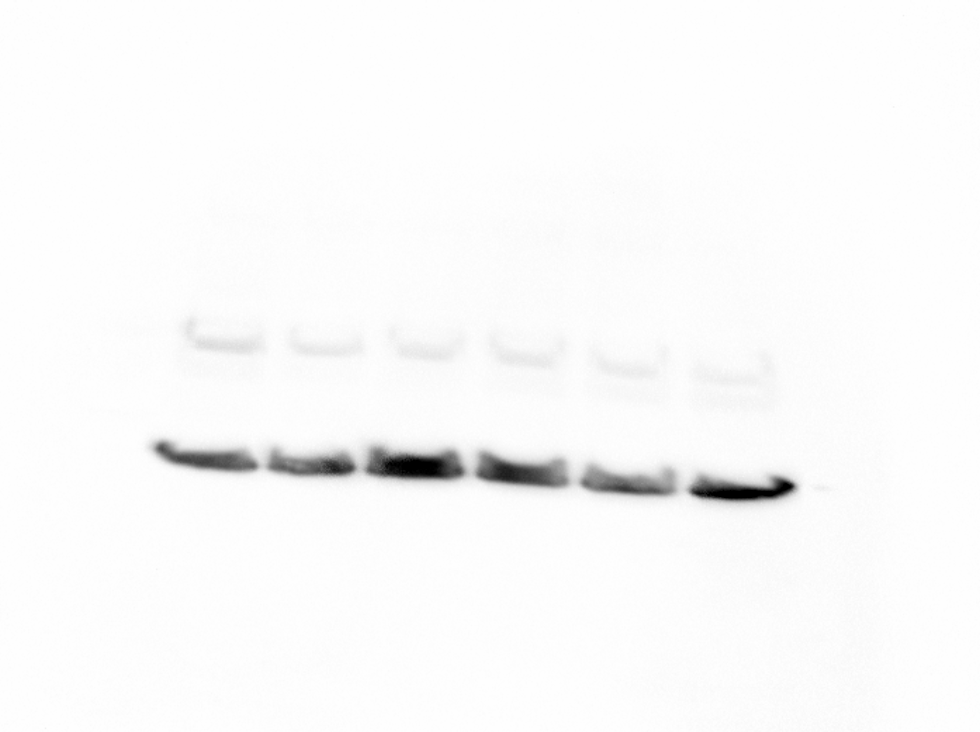

Supplement: S8 Data — (ZIP) [file pone.0157483.s012.zip › NFKB Blots/GAPDH/2.tif]

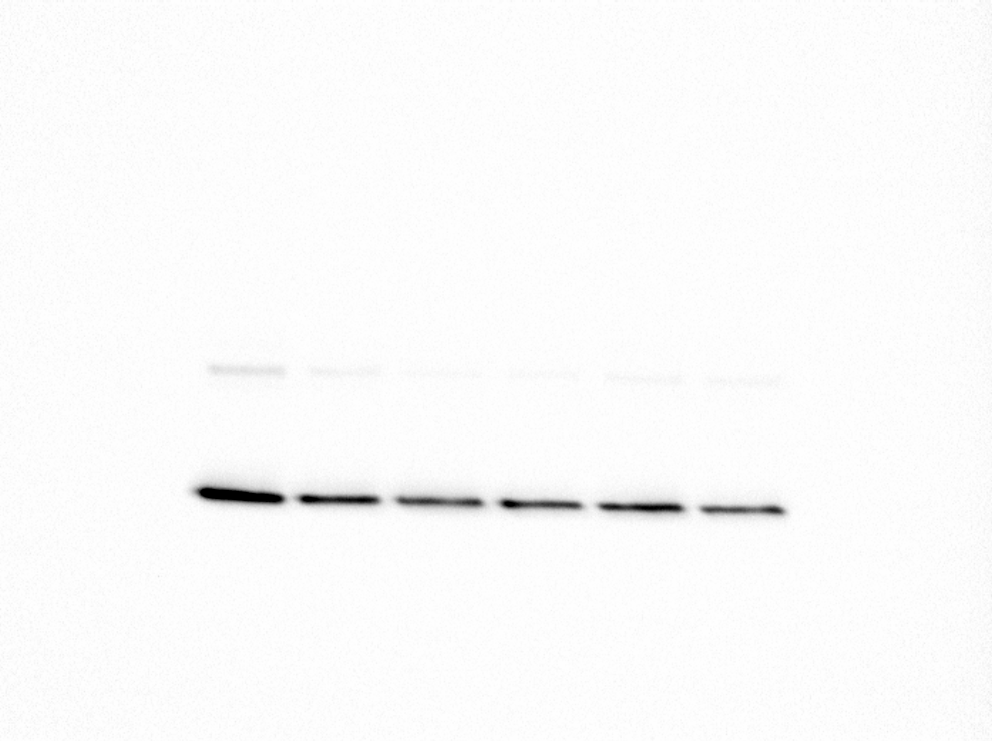

Supplement: S8 Data — (ZIP) [file pone.0157483.s012.zip › NFKB Blots/GAPDH/Blot 3.tif]

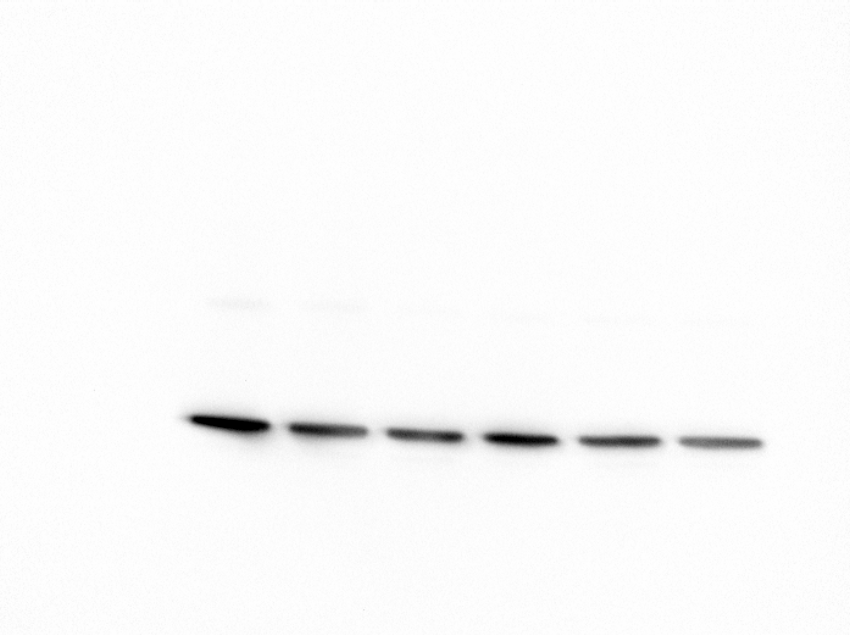

Supplement: S8 Data — (ZIP) [file pone.0157483.s012.zip › NFKB Blots/GAPDH/Jarred Griffin 2014-03-14 11hr 40min_Exposure_20.0sec.tif]

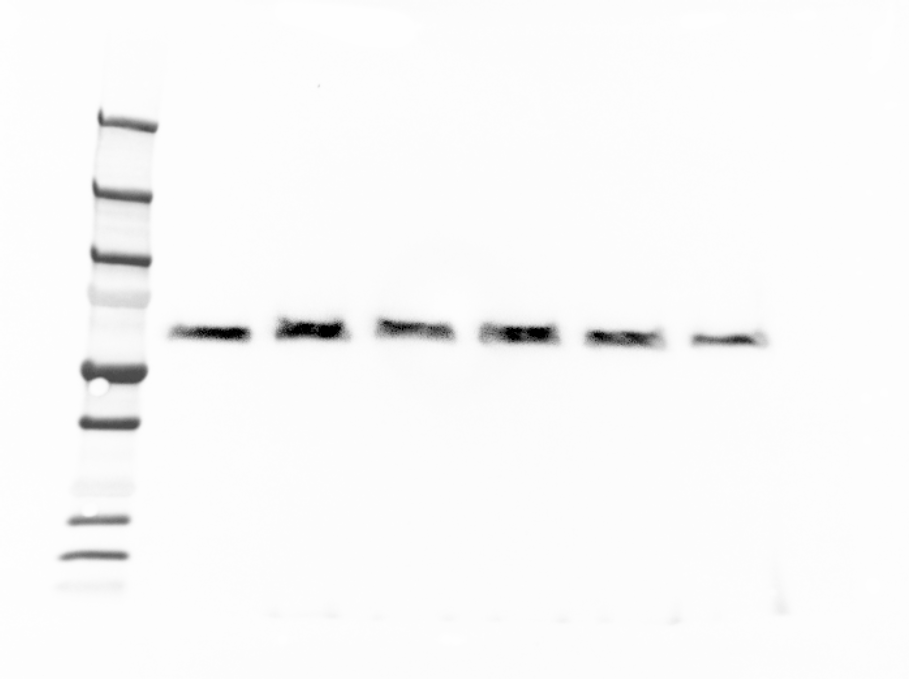

Supplement: S8 Data — (ZIP) [file pone.0157483.s012.zip › NFKB Blots/NFKB/Blot 1.tif]

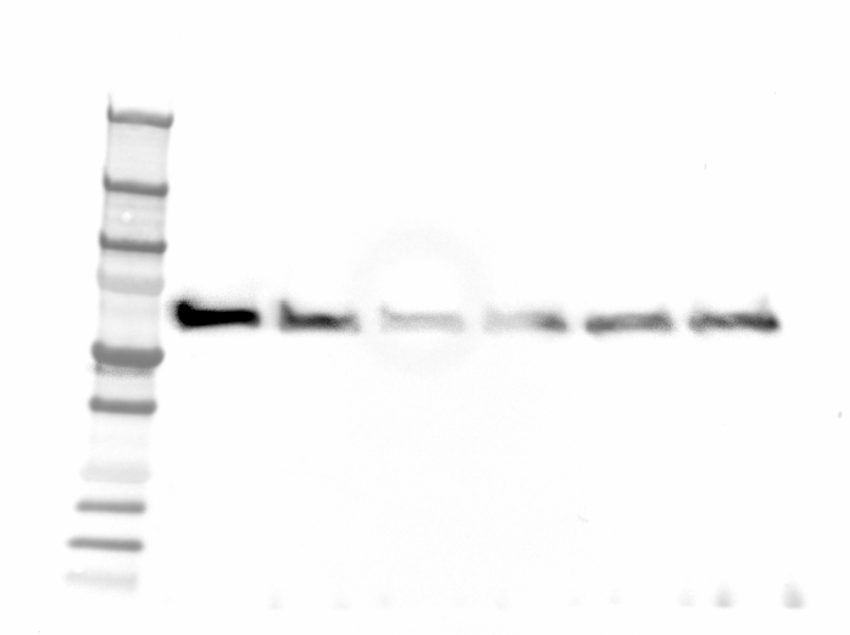

Supplement: S8 Data — (ZIP) [file pone.0157483.s012.zip › NFKB Blots/NFKB/Blot 2.tif]

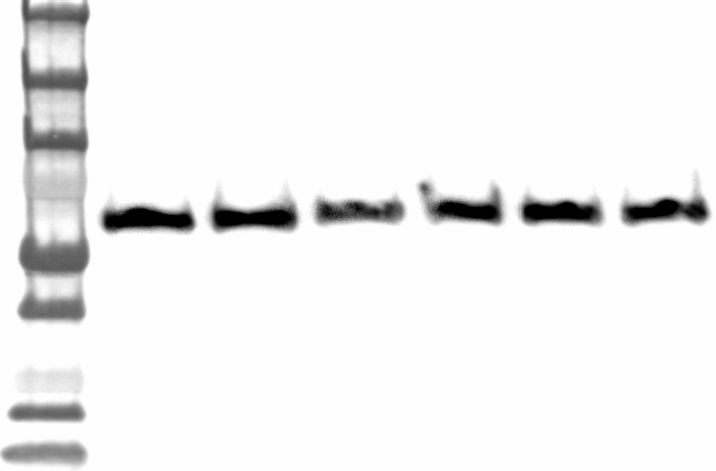

Supplement: S8 Data — (ZIP) [file pone.0157483.s012.zip › NFKB Blots/NFKB/Blot 3.tif]

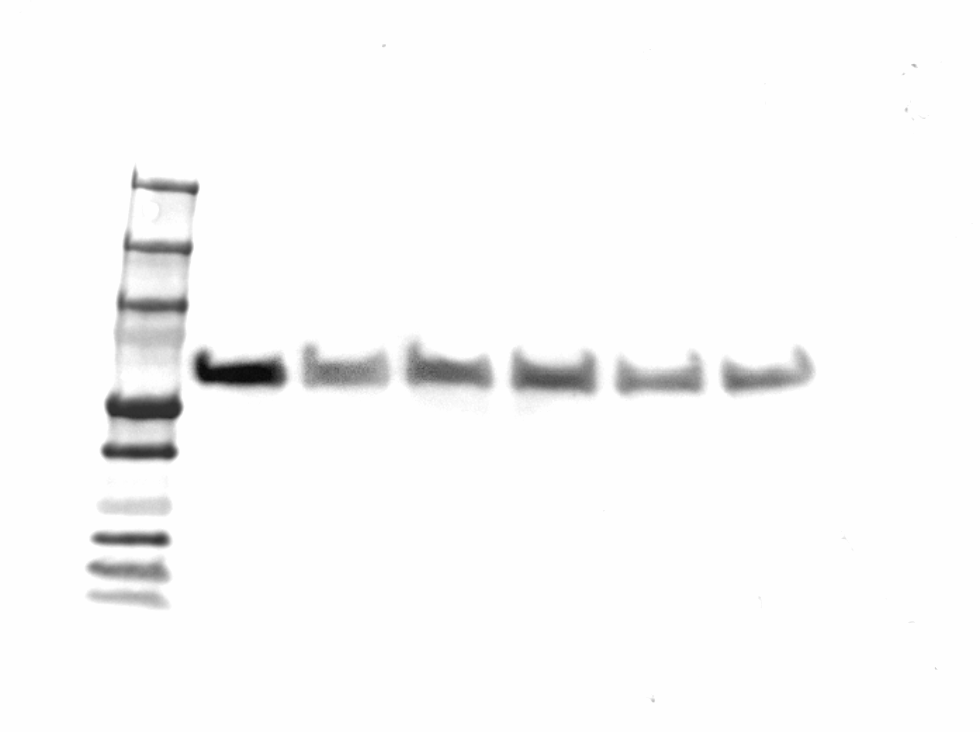

Supplement: S8 Data — (ZIP) [file pone.0157483.s012.zip › NFKB Blots/NFKB/Blot 4.tif]

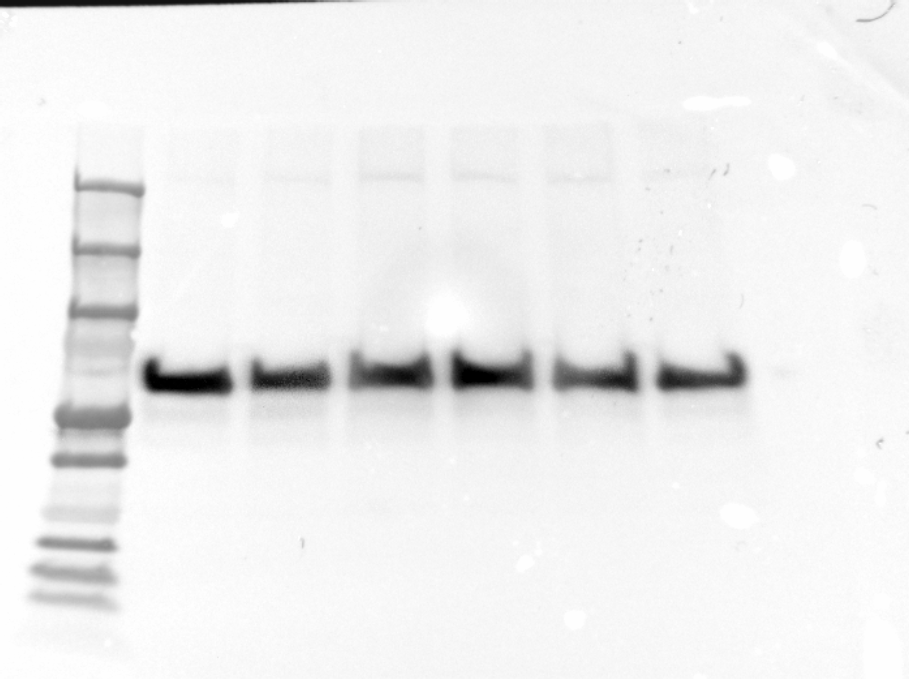

Supplement: S8 Data — (ZIP) [file pone.0157483.s012.zip › NFKB Blots/NFKB/Blot 5.tif]

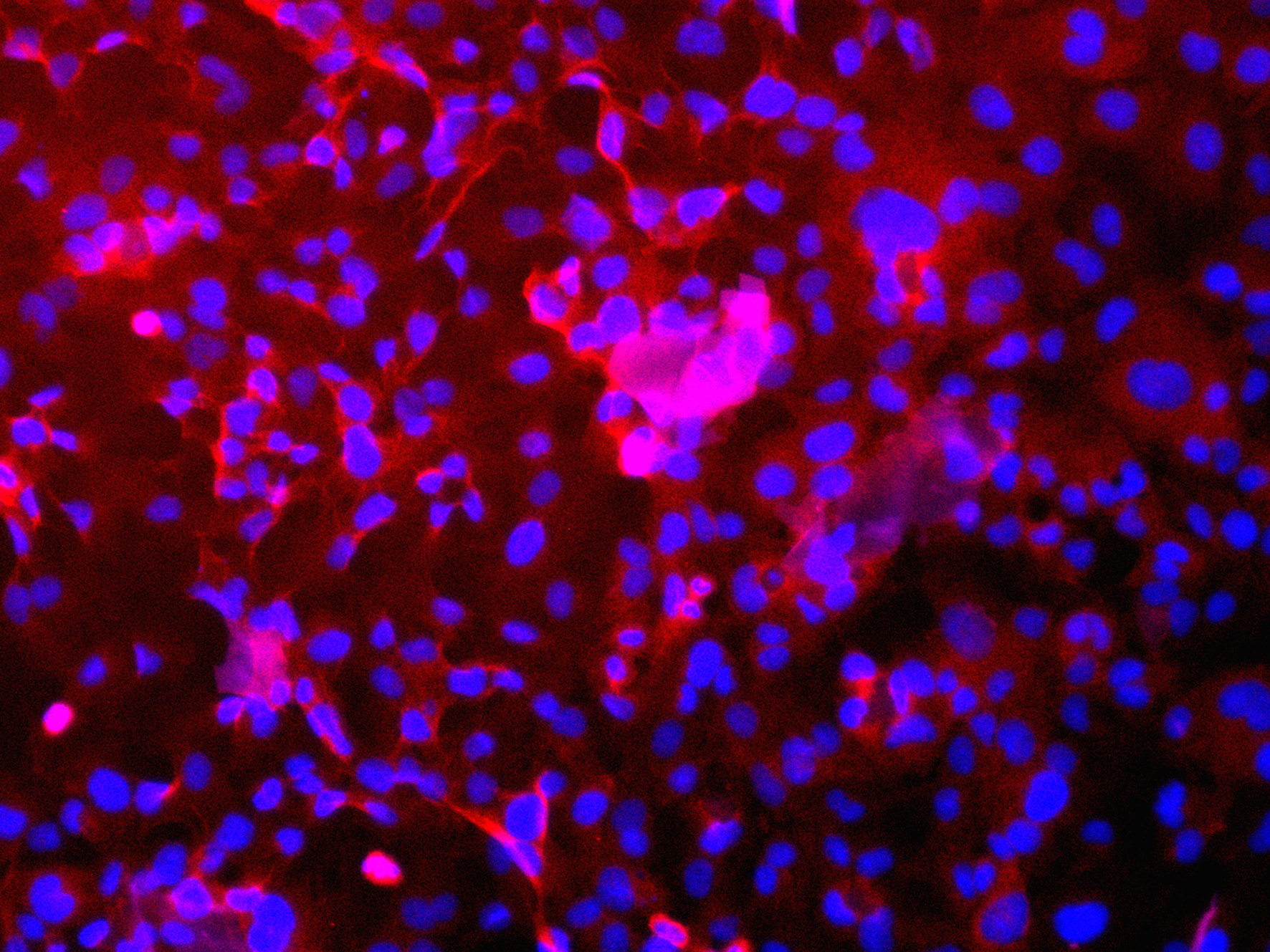

Supplement: S9 Data — (ZIP) [file pone.0157483.s013.zip › NFKB Images/AB/AB.tif]

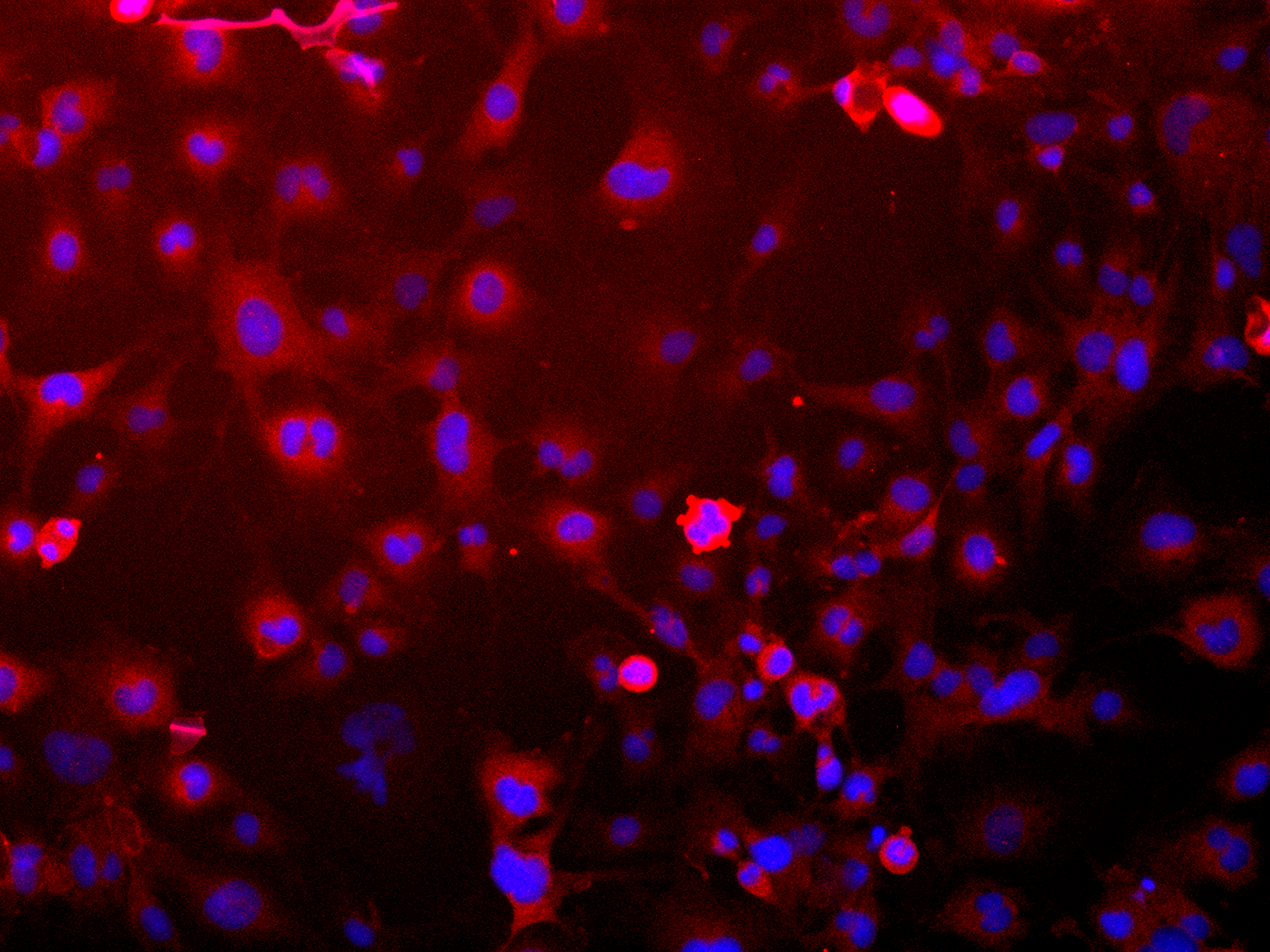

Supplement: S9 Data — (ZIP) [file pone.0157483.s013.zip › NFKB Images/AB + Simva/AB + Simva.tif]

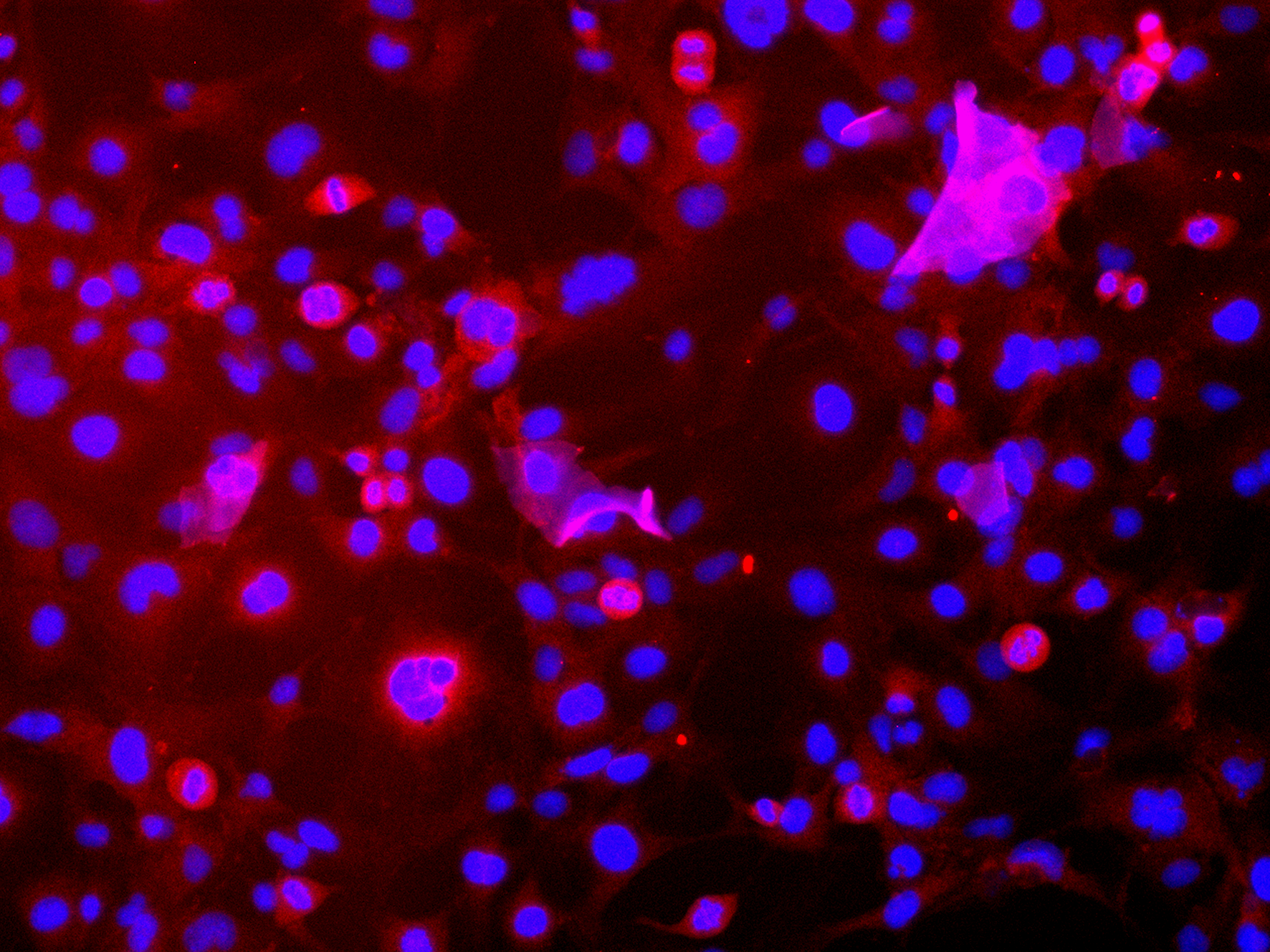

Supplement: S9 Data — (ZIP) [file pone.0157483.s013.zip › NFKB Images/AB+Lova/RGB.tif]

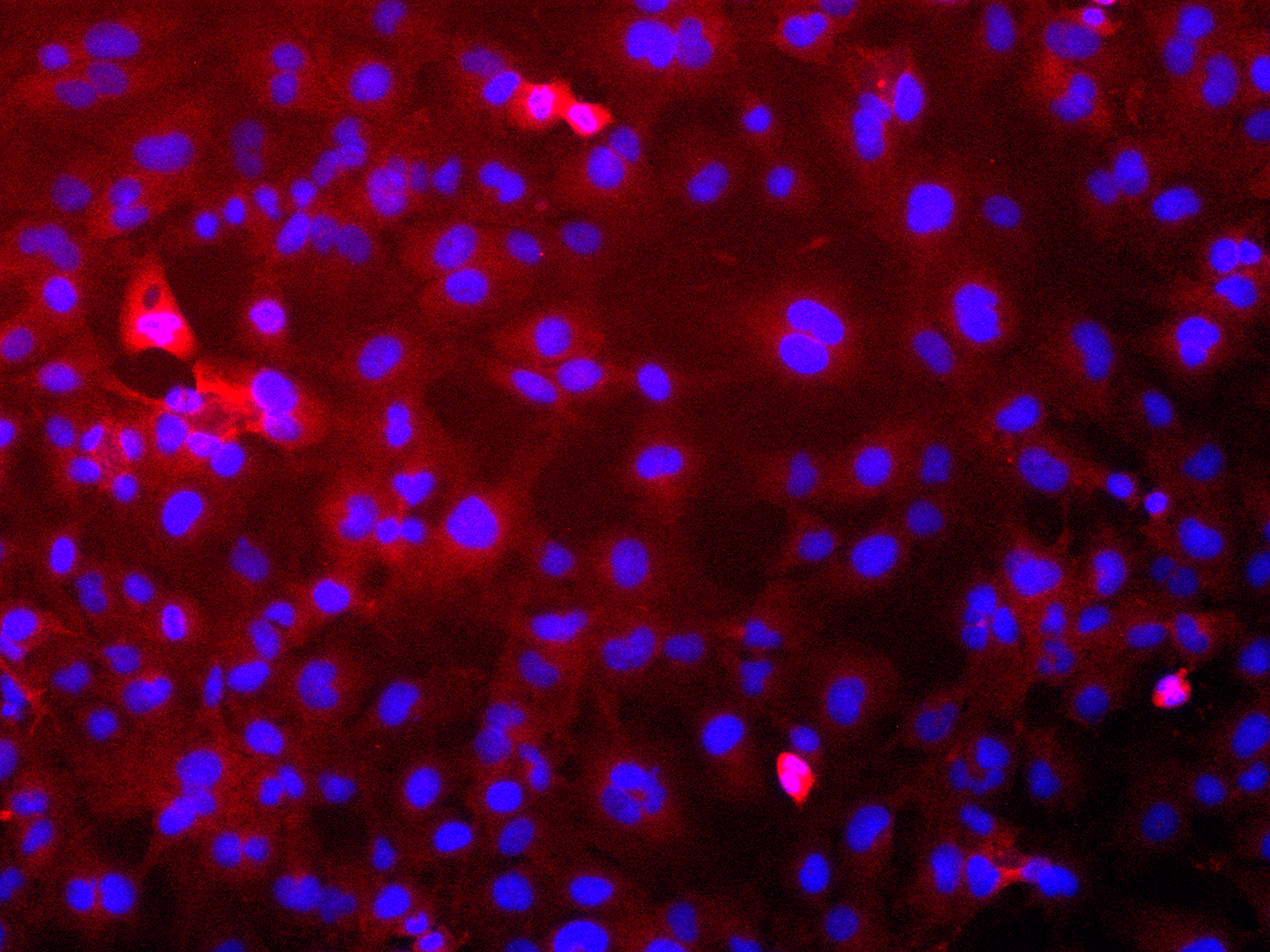

Supplement: S9 Data — (ZIP) [file pone.0157483.s013.zip › NFKB Images/Control/Control .tif]

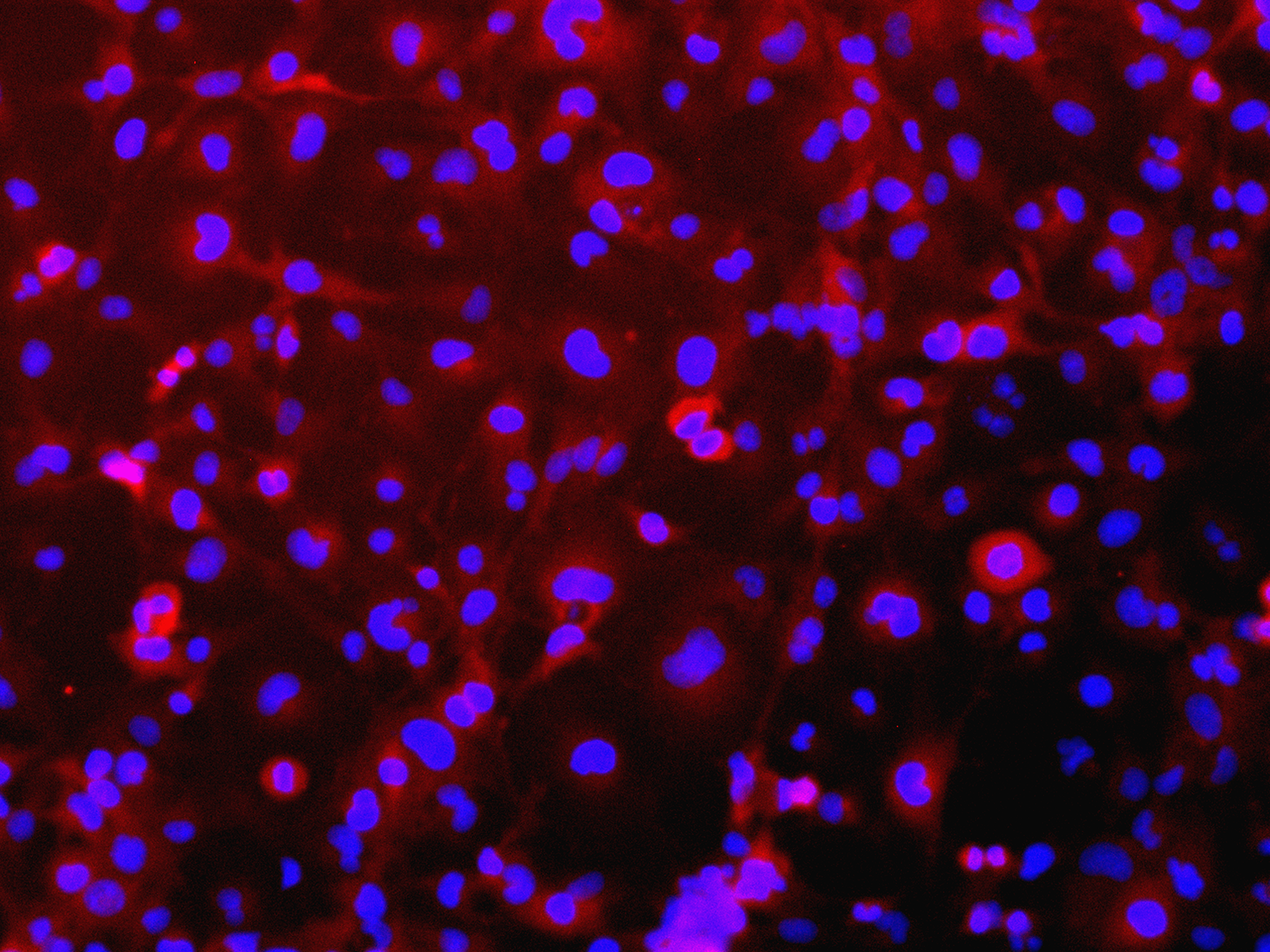

Supplement: S9 Data — (ZIP) [file pone.0157483.s013.zip › NFKB Images/Lovastatin/Lovastatin.tif]

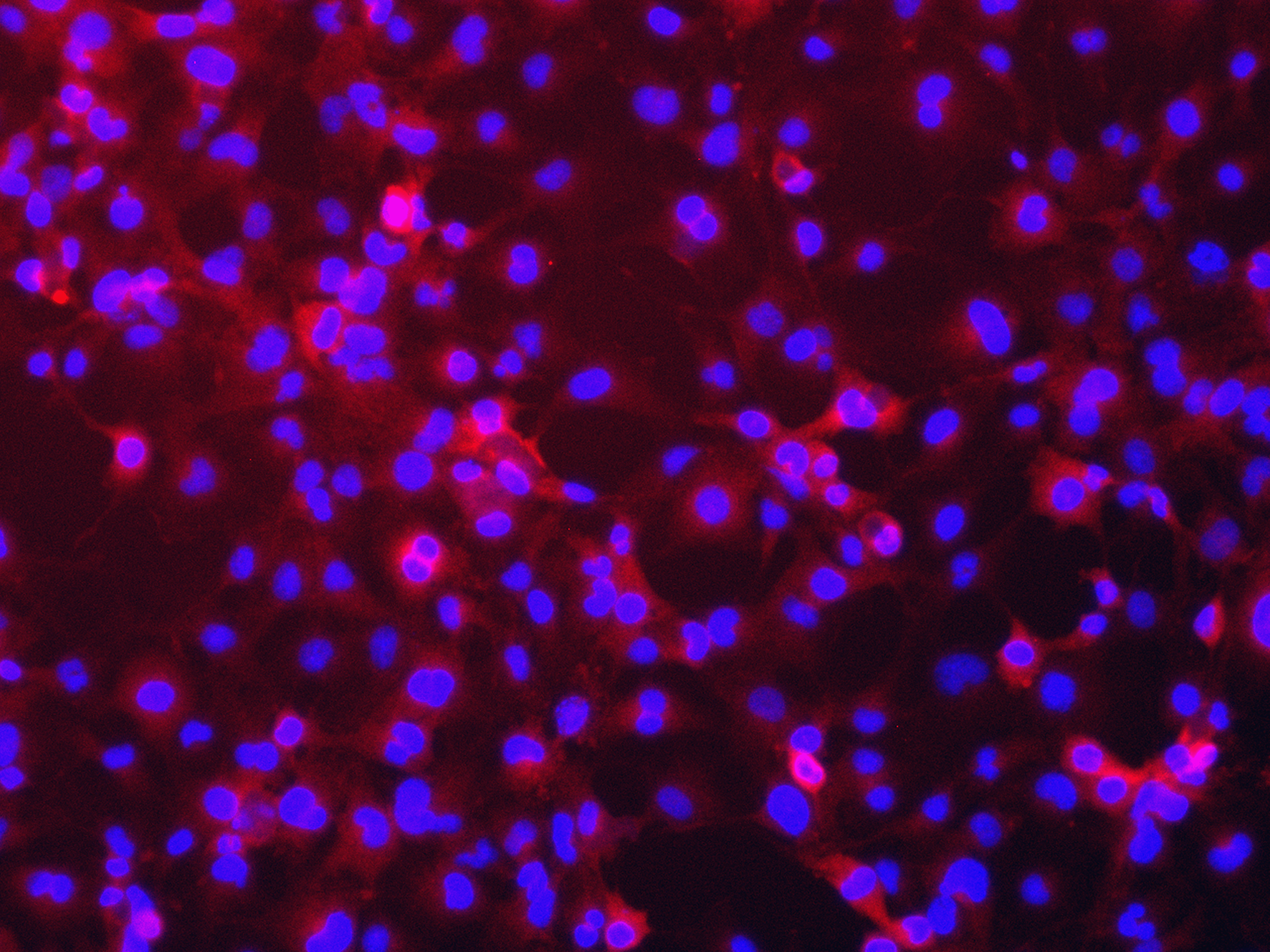

Supplement: S9 Data — (ZIP) [file pone.0157483.s013.zip › NFKB Images/Simvastatin/Simvastatin.tif]
